# Supplementary material for: Distinct Rabi splitting in confined systems of MoSe2 monolayers and (Ga,In)As quantum wells
Source: Nat Commun. 2025 Aug 29;16:8109. doi: 10.1038/s41467-025-63098-7 (PMC12397422; doi:10.1038/s41467-025-63098-7)
Supplement: Supplementary file 1 — Supplementary Information [file 41467_2025_63098_MOESM1_ESM.pdf]

Supplemental Material: Distinct Rabi splitting in confined systems of $MoSe_2$ Monolayers and (Ga,In)As Quantum Wells

Felix Schäfer*, Markus Stein†, Daniel Anders, Isabel Müller, Florian Dobener, and Sangam Chatterjee
Institute of Experimental Physics I and Center for Materials Research (LaMa), Justus-Liebig-University Giessen, Heinrich-Buff-Ring 16, D-35392 Giessen, Germany

Henry Mittenzwey*
Nichtlineare Optik und Quantenelektronik, Institut für Physik und Astronomie (IFPA), Technische Universität Berlin, D-10623 Berlin, Germany

Oliver Voigt, Lara Greten, and Andreas Knorr
Nichtlineare Optik und Quantenelektronik, Institut für Physik und Astronomie (IFPA), Fachgruppe Theoretische Physik, Technische Universität Berlin, D-10623 Berlin, Germany

Marzia Cuccu and Alexey Chernikov
Dresden Integrated Center for Applied Physics and Photonic Materials (IAPP) and Würzburg-Dresden Cluster of Excellence ct.qmat, Technische Universität Dresden, D-01062 Dresden, Germany

Christian Fuchs and Kerstin Volz
Structure & Technology Research Laboratory (WZMW), Philipps-University Marburg, Hans-Meerwein-Straße 6, D-35032 Marburg, Germany

Kenji Watanabe
Research Center for Electronic and Optical Materials, National Institute for Materials Science, 1-1 Namiki, Tsukuba 305-0044, Japan

Takashi Taniguchi
Research Center for Materials Nanoarchitectonics, National Institute for Materials Science, 1-1 Namiki, Tsukuba 305-0044, Japan
(Dated: July 31, 2025)

* These two authors contributed equally

† markus.stein@exp1.physik.uni-giessen.de

ABSTRACT

This supplementary material provides additional experimental and theoretical details supporting the main manuscript. In the first part, we present experimental procedures used to characterize the $MoSe_2$ monolayer and optimize the excitation conditions for observing symmetric Rabi splitting. Hyperspatial photoluminescence mapping was performed at cryogenic temperatures to identify high-quality regions of the sample with minimal strain and disorder. By systematically varying the central energy and spectral width of the pump pulses, we identify the optimal excitation parameters, which are then used for all reported measurements. We further present a direct comparison of the transient absorption dynamics in $MoSe_2$ and (Ga,In)As multiple quantum wells, highlighting their distinct excitation-dependent features and many-body response through two-dimensional false-color maps. In the second part, we outline the microscopic theoretical framework employed to model the exciton dynamics in both material systems. The theory is based on a many-body approach using the Heisenberg equations of motion, incorporating an exciton expansion to capture light–matter and Coulomb interactions. It includes the equations of motion for excitonic transitions, occupations, biexcitons, and six-particle correlations, as well as treatments of phonon-assisted exciton formation and screened Coulomb interactions. Numerical simulation parameters are explicitly provided, alongside analytical results elucidating Rabi-splitting behavior.

Contents

Abstract 1

Experimental Details 2
Hyperspatial Photoluminescence mapping of the TMDC Monolayer 2
Different pump pulses 3
Comparison TMDC Monolayer and MQW 3

Theory 5
Equations of Motion 5
Exciton-Exciton Interaction Matrix Elements 8
Optical Observable 8
Screened Coulomb Potential 9
Linear Absorption 11
Analytical Derivation of the Rabi Splitting 12
Estimating the Optical Field Strength at a Given Laser Power 14
Rescaling the Exciton-Exciton Interaction Strength in a GaAs QW 15
Quantifying the Exciton-Phonon Interaction in a GaAs QW 16
Origin of the Asymmetric Rabi Splitting in a $MoSe_2$ Monolayer 18
Simulation Parameters 20

References 21

EXPERIMENTAL DETAILS

Hyperspatial Photoluminescence mapping of the TMDC Monolayer

In our study we performed hyperspatial photoluminescence (PL) mapping of the $MoSe_2$ monolayer using low-power continuous-wave excitation at liquid helium temperatures. The extracted energy of the neutral exciton for the studied $MoSe_2$ monolayer is presented in Supplementary Figure 1 (a). The map shows regions with strong fluctuations of the exciton energy due to both substrate-induced strain and formation of bubbles. Most importantly, it allows us to

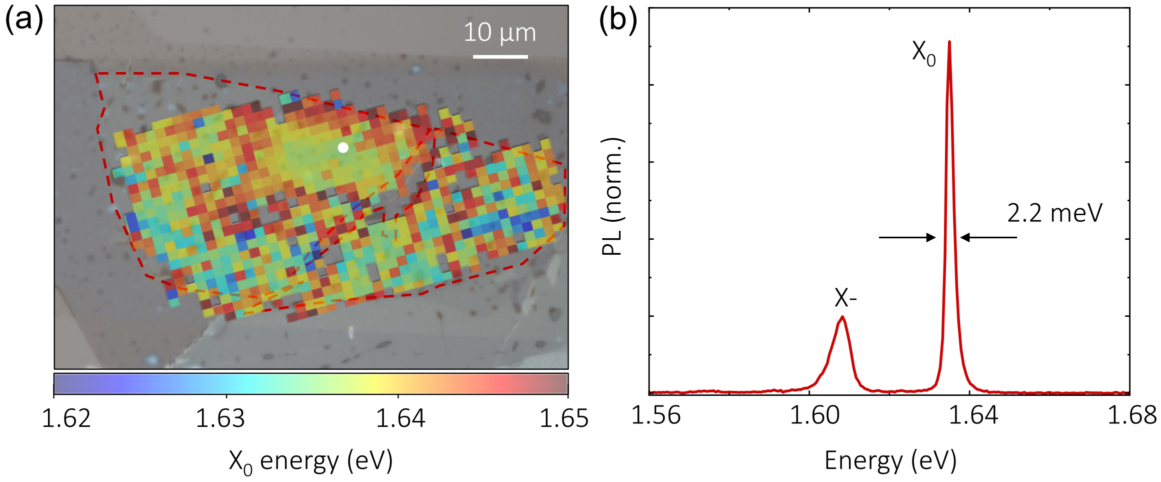

Supplementary Figure 1: (a) Spatially-resolved resonance energy of the neutral exciton resonance (X_0) extracted from hyperspatial photoluminescence mapping. A continuous-wave laser with 532 nm wavelength was used for excitation with a low power density of 90 W/cm^2 focused on a 1 µm spot. The step size for scanning was set to 2 µm and the measurements were performed at the heat-sink temperature of 5 K. (b) Characteristic PL spectrum obtained in a region with smooth energy profile, as indicated in panel (b). The spectrum shows characteristic features of neutral and charged excitons with narrow line-widths as expected for high-quality hBN-encapsulated samples.

identify sufficiently large areas on a scale of 10 µm with smooth potential profiles. These areas feature characteristic optical features of high-quality $MoSe_2$ with narrow spectral lines due to suppressed disorder, see example in Supplementary Figure 1 (a). All subsequent measurements using pump-probe spectroscopy are performed on such areas with good interlayer contact in absence of bubbles and strain fluctuations.

Different pump pulses

We tested a range of pump pulse parameters to optimize conditions for achieving a symmetric Rabi splitting in the $MoSe_2$ monolayer. Specifically, we varied both the central energy and the spectral width of the excitation pulses. Supplementary Figure 2 displays a selection of pump energies (indicated by gray shaded regions) alongside the corresponding nonlinear absorption spectra, recorded at identical time delays and pump powers. The black vertical line denotes the exciton resonance of the unexcited sample. Among the tested configurations, we found that a spectrally narrow excitation pulse, slightly blue-shifted from the exciton resonance (green line), produced the most symmetric splitting of the absorption feature. Based on this optimization, we selected this excitation condition for all measurements on the $MoSe_2$ monolayer that are shown in the main manuscript.

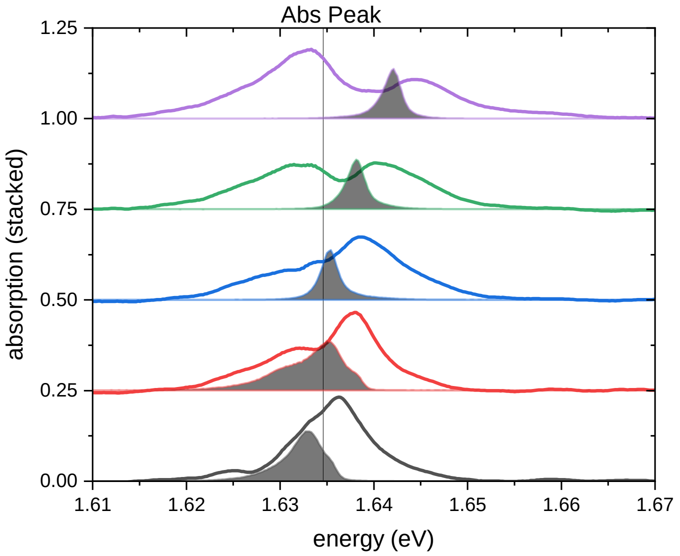

Supplementary Figure 2: Effect of pump pulse energy and spectral width on the excited-state absorption in $MoSe_2$. The gray shaded areas represent the spectral profiles of the pump pulses, each applied at the same delay time and excitation fluence. The overlaid colored lines show the corresponding nonlinear absorption spectra.

Comparison TMDC Monolayer and MQW

Supplementary Figure 3 presents two-dimensional false-color plots of the absorption dynamics in a $MoSe_2$ monolayer (top panels) and a (Ga,In)As multiple quantum well (MQW) structure (bottom panels). The horizontal axis corresponds to the photon energy, while the vertical axis shows the pump–probe delay, with zero delay indicating temporal overlap of the excitation and probe pulses. The color scale represents the absorption signal αL. For the **$MoSe_2$ monolayer**, we compare the transient absorption response under weak (16 µJ/cm^2, left) and strong (63 µJ/cm^2, right) excitation conditions. At negative time delays, the linear absorption spectrum of the unperturbed $1s$ exciton is visible. Under weak excitation, a symmetric splitting into a doublet appears at time zero, marking the onset of Rabi splitting of the exciton resonance. Once the pump and probe pulses no longer temporally overlap (beyond ∼0.5 ps), the doublet collapses into a broadened and bleached single resonance. At higher excitation fluences, the dynamics change qualitatively: the splitting becomes asymmetric, and a pronounced blue shift of the main absorption peak emerges at zero delay. With increasing delay, the high-energy absorption peak shifts back toward the unperturbed transition energy, eventually forming a broadened, weakened exciton resonance. In contrast, the **(Ga,In)As MQW** panels (bottom) show analogous yet distinctly different dynamics, reflecting the altered balance between Coulomb

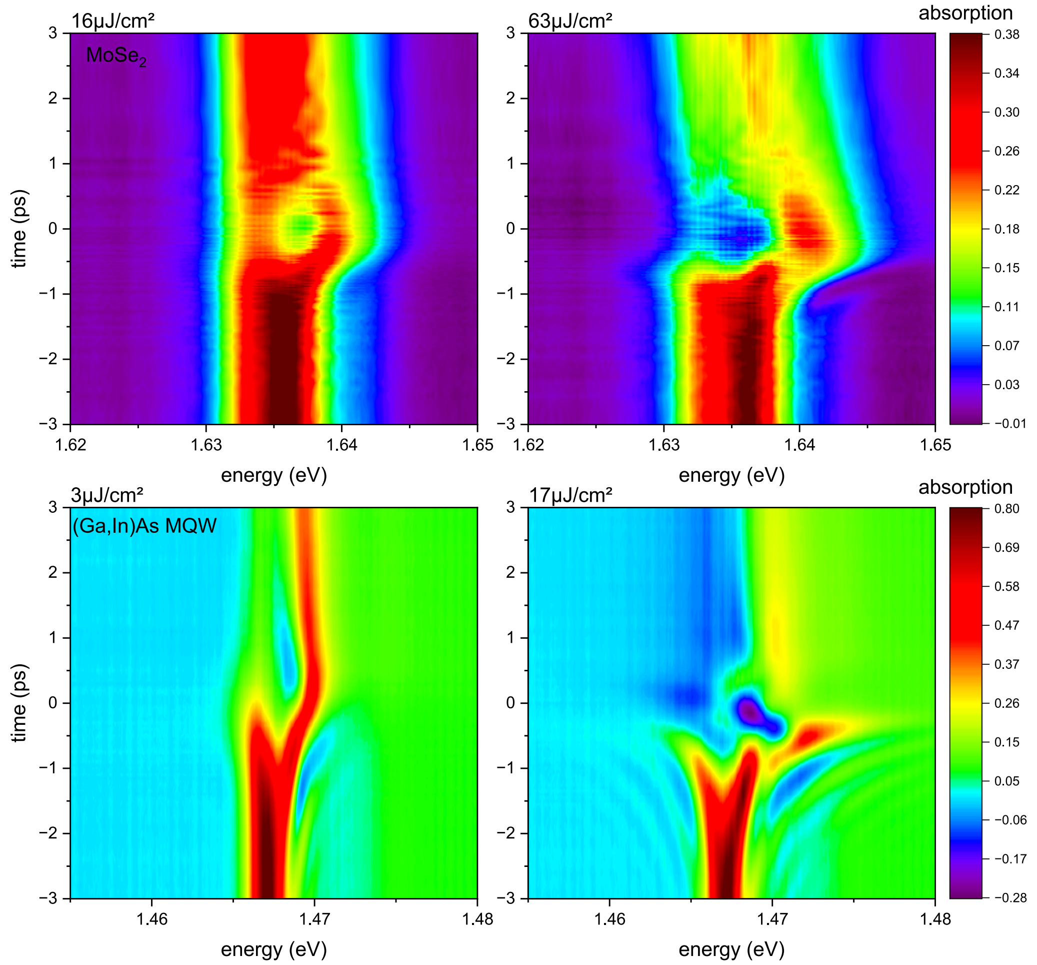

Supplementary Figure 3: Two-dimensional false-color maps of transient absorption dynamics in a $MoSe_2$ monolayer (top panels) and a (Ga,In)As MQW structure (bottom panels) under weak (left) and strong (right) excitation. The vertical axis shows the pump-probe delay (with temporal overlap at 0 ps), and the horizontal axis represents photon energy.

interaction and light-matter coupling. Under weak excitation (left), a transient Rabi splitting is observed near 0 ps, followed by a smooth evolution into a single, blue-shifted resonance at later times. Under strong excitation (right), the system enters a non-perturbative regime. A triplet structure emerges at early delays (∼–0.5 ps), which evolves into coherent optical gain at 1.469 eV and a strongly blue-shifted resonance at 1.473 eV at temporal overlap. Beyond 0.5 ps, these features merge into a highly bleached peak near 1.471 eV that blends into the continuum absorption. Together, these measurements reveal the excitation-dependent evolution of light-dressed exciton resonances and highlight key many-body phenomena such as Coulomb screening, gain, and incoherent carrier dynamics. Although the absolute energy scales differ between the $MoSe_2$ and MQW systems due to their material-specific band structures, the temporal windows are identical across all panels, enabling a direct comparison of their ultrafast light-matter interaction dynamics.

THEORY

Equations of Motion

The equations of motion of the excitonic transitions P read:

$$
\begin{aligned}
\mathrm{i}\hbar\partial_t P^{\xi} =& (E_{1s} - \mathrm{i}\hbar\gamma_{\mathrm{nrad}})P^{\xi} - \sum_{\mathbf{q}} \varphi^*_{\mathbf{q}} \hbar\Omega^{cv,\xi} + 2\sum_{\mathbf{q}} \varphi^*_{\mathbf{q}} \hbar\Omega^{cv,\xi} \left|\varphi_{\mathbf{q}}\right|^2 \left(N^{\xi} + \left|P^{\xi}\right|^2\right) \\
&+ 2\sum_{\mathbf{q},\mathbf{k}} V_{\mathbf{q}-\mathbf{k}} \left|\varphi_{\mathbf{q}}\right|^2 \left(\varphi^*_{\mathbf{q}}\varphi_{\mathbf{k}} - |\varphi_{\mathbf{k}}|^2\right) \left(2N^{\xi} + \left|P^{\xi}\right|^2\right) P^{\xi} + 2\sum_{\mathbf{q},\mathbf{k}} V^{\xi,\xi}_{\mathrm{X},\mathbf{q}-\mathbf{k}} \left|\varphi_{\mathbf{q}}\right|^2 \left|\varphi_{\mathbf{k}}\right|^2 \left(2N^{\xi} + \left|P^{\xi}\right|^2\right) P^{\xi} \\
&+ \sum_{\pm,\zeta,\mathbf{Q},\xi'} \left(\pm W_{1,\mathbf{Q},\mathbf{0}} - W_{2,\mathbf{Q},\mathbf{0}}\right)^* \Phi^{\mathrm{R}}_{\pm,\zeta,\mathbf{Q}} \left(B^{\xi,\xi'}_{\pm,\zeta} P^{*,\xi'} + Z^{\xi',\xi,\xi'}_{\pm,\zeta}\right) \\
&+ \sum_{\pm,\zeta,\mathbf{Q},\xi'} \left(\pm W_{\mathrm{X},1,\mathbf{Q},\mathbf{0}} - W_{\mathrm{X},2,\mathbf{Q},\mathbf{0}}\right)^* \Phi^{\mathrm{R}}_{\pm,\zeta,\mathbf{Q}} \left(B^{\xi,\xi'}_{\pm,\zeta} P^{*,\xi} + Z^{\xi,\xi,\xi'}_{\pm,\zeta}\right).
\end{aligned}
\tag{6}
$$

In case of the $MoSe_2$ monolayer, ξ denotes the valley and spin of the optically allowed energy-degenerate $K,\uparrow,K,\uparrow$- or $K',\downarrow,K',\downarrow$-transitions [1]. In case of the MQW sample, ξ denotes the optically allowed energy-degenerate heavy hole $\left(\frac{3}{2},\frac{1}{2}\right)$- or $\left(-\frac{3}{2},-\frac{1}{2}\right)$-transition at the Γ valley [2, 3]. E_{1s} is the $1s$ excitonic energy, γ_{nrad} is the nonradiative dephasing, $\hbar\Omega^{cv} = \mathbf{d}^{cv,\xi}\cdot\mathbf{E}$ is the Rabi energy with transition dipole moment $\mathbf{d}^{cv,\xi}$ and optical renormalized field $\mathbf{E}$, which is obtained by solving Maxwell's equations in a plane wave geometry [4], given by:

$$
\mathbf{E} = \mathbf{E}_0 - \frac{1}{2\epsilon_0 c_0 n_{\mathrm{ref}}}\partial_t \mathbf{P}. \tag{7}
$$

Here, $\mathbf{E}_0$ is the incident optical pulse with center frequency ω_0, given by:

$$
\mathbf{E}_0(t) = \frac{E_0}{\sqrt{2\pi}\sigma} \mathrm{e}^{-\frac{t^2}{2\sigma^2}} \mathrm{Re}\left(\left(E_+\mathbf{e}_+ + E_-\mathbf{e}_-\right)\mathrm{e}^{-\mathrm{i}\omega_0 t}\right), \tag{8}
$$

where $\frac{E_0}{\sqrt{2\pi}\sigma}$ is the time-normalized amplitude of the optical pulse with duration σ, which is related to the intensity full width at half maximum: $\mathrm{FWHM} = 2\sqrt{\ln(2)}\sigma$. $\mathbf{e}_\pm = \frac{1}{\sqrt{2}}\begin{pmatrix}1\\ \pm\mathrm{i}\end{pmatrix}$ are the Jones vectors and $E_\pm$ are the polarization coefficients in circular basis, which read: $E_\pm = \frac{1}{\sqrt{2}}\left(E_x \mp \mathrm{i}E_y \mathrm{e}^{\mathrm{i}\delta}\right)$, with the coefficients in the cartesian basis $E_{x/y}$ and phase difference δ. Moreover, in Eq. (7), ϵ_0 is the vacuum permittivity, c_0 the velocity of light in vacuum, $n_{\mathrm{ref}} = \frac{1}{2}\left(\sqrt{\epsilon_{1,\infty,\|}} + \sqrt{\epsilon_{2,\infty,\|}}\right)$ is the refractive index averaged over substrate and superstrate material with optical in-plane dielectric constants $\epsilon_{1,\infty,\|}$ and $\epsilon_{2,\infty,\|}$, respectively, and $\mathbf{P}$ is the macroscopic polarization, which reads:

$$
\mathbf{P} = \frac{1}{\mathcal{A}}\sum_{\mathbf{q},\xi}\left(\varphi_{\mathbf{q}}\mathbf{d}^{vc,\xi}P^{\xi} + \mathrm{c.c.}\right). \tag{9}
$$

Here, $\mathcal{A}$ is the area of the semiconductor.
Further, in Eq. (6), $V_{\mathbf{q}}$ is the quantum-confined Coulomb potential, which is provided in Eq. (36) in Sec. *Screened Coulomb Potential.* $V^{\xi,\xi'}_{\mathrm{X},\mathbf{q}}$ is the exchange interaction potential, given by:

$$
V^{\xi,\xi'}_{\mathrm{X},\mathbf{q}} = V_{\mathrm{SR}}\delta_{\xi,\xi'} + V_{\mathbf{q}}\frac{1}{e^2}\left(\mathbf{q}\cdot\mathbf{d}^{vc,\xi}\right)\left(\mathbf{q}\cdot\mathbf{d}^{cv,\xi'}\right), \tag{10}
$$

where the first term is the short-range potential and the second term is the long-range potential. W_1, W_2, $W_{\mathrm{X},1}$ and $W_{\mathrm{X},2}$ are the direct and exchange exciton-exciton interaction matrix elements, given in Eq. (20), Eq. (21), Eq. (22) and Eq. (23), respectively.

$\varphi_{\mathbf{q}}$ is the excitonic wave function, which solves the Wannier equation:

$$
\left(E_{\mathrm{gap}} + \frac{\hbar^2\mathbf{q}^2}{2}\left(\frac{1}{m_h} + \frac{1}{m_e}\right)\right)\varphi_{\mu,\mathbf{q}} - \sum_{\mathbf{q}'} V_{\mathbf{q}-\mathbf{q}'}\varphi_{\mu,\mathbf{q}'} = E_\mu\varphi_{\mu,\mathbf{q}}, \tag{11}
$$

which describes the relative motion of electron and hole with masses $m_{e/h}$. E_{gap} is the renormalized free-particle band gap and E_μ are the excitonic energies with quantum number μ. The two-exciton wave function $\Phi^{\text{R}}_{\pm,\zeta,\mathbf{Q}}$ solves the two-exciton Schrödinger equation:

$$\left(E_{1s}+E_{1s}+\frac{\hbar^2\mathbf{K}^2}{2}\left(\frac{1}{M}+\frac{1}{M}\right)\right)\Phi^{\text{R}}_{\pm,\zeta;\mathbf{K}}-\sum_{\rho,\lambda,\mathbf{Q}}\left(S^{-1}_{\pm}\right)_{1s,1s,\mathbf{K};\rho,\lambda,\mathbf{Q}}\sum_{\mathbf{Q}'}\left(W_{1,\rho,\lambda,\mathbf{Q};1s,1s,\mathbf{Q}'}\mp W_{2,\rho,\lambda,\mathbf{Q};1s,1s,\mathbf{Q}'}\right)\Phi^{\text{R}}_{\pm,\zeta;\mathbf{Q}'} = E^{\text{XX}}_{\pm,\zeta}\Phi^{\text{R}}_{\pm,\zeta;\mathbf{K}}, \quad (12)$$

which describes the relative motion of two excitons with exciton masses $M=m_e+m_h$. Here, $E^{\text{XX}}_{\pm,\zeta}$ are the two-exciton energies with two-exciton quantum number ζ, which, in the "−" spin configuration, contain one bound state and a continuum and, in the "+" configuration, contain only continuum states. $(S_\pm)_{\mu,\nu,\mathbf{K};\rho,\lambda,\mathbf{Q}}$ is the projection matrix, cf. Eq. (19), which emerges due to the expansion in symmetric/antisymmetric spin states [5, 6], and $W_{1,\rho,\lambda,\mathbf{Q};1s,1s,\mathbf{Q}'}$ and $W_{2,\rho,\lambda,\mathbf{Q};1s,1s,\mathbf{Q}'}$ are the exciton-exciton interaction matrices, cf. Eq. (20) and Eq. (21), respectively. ρ, λ represent excitonic states. We note, that we disregard the biexciton fine structure [7], which can alter the biexciton binding energies depending on the exchange interaction strength [8].

The equations of motion of the excitonic occupations N in the $N_{\mathbf{Q}}\approx\delta_{\mathbf{Q},\mathbf{0}}N$-approximation read:

$$\text{i}\hbar\partial_t N^\xi = \text{i}\hbar\partial_t N^\xi\big|_{\text{opt+XX}} + \text{i}\hbar\partial_t N^\xi\big|_{\text{phon}}. \quad (13)$$

The optical and exciton-exciton contributions $\text{i}\hbar\partial_t N^\xi\big|_{\text{opt+XX}}$ read:

$$\begin{aligned}\text{i}\hbar\partial_t N^\xi\big|_{\text{opt+XX}} =& \hbar\Omega^{vc,\xi}\sum_{\mathbf{q}}\varphi_{\mathbf{q}}\sum_{\mathbf{Q}}\left|\varphi_{\mathbf{Q}}\right|^2\left(\left|\varphi_{\alpha\mathbf{q}+\beta\mathbf{Q}}\right|^2+\left|\varphi_{\alpha\mathbf{Q}+\beta\mathbf{q}}\right|^2\right)P^\xi\left|P^{*\xi}\right|^2\\ &+\hbar\Omega^{vc}\sum_{\mathbf{q}}\varphi_{\mathbf{q}}\left(\sum_{\mathbf{Q}}\left|\varphi_{\mathbf{Q}}\right|^2\left(\left|\varphi_{\alpha\mathbf{q}+\beta\mathbf{Q}}\right|^2+\left|\varphi_{\alpha\mathbf{Q}+\beta\mathbf{q}}\right|^2\right)-\left|\varphi_{\mathbf{q}}\right|^2\right)2P^\xi N^\xi\\ &+\hbar\Omega^{vc,\xi}\sum_{\zeta,\mathbf{Q}}D_{\mathbf{Q}}\Phi^{\text{R}}_{+,\zeta,\mathbf{Q}}\left(P^{*\xi}B^{\xi,\xi}_{+,\zeta}+Z^{\xi,\xi,\xi}_{+,\zeta}\right)\\ &-\sum_{\zeta,\mathbf{Q}}W_{B,\mathbf{Q}}\Phi^{\text{R}}_{+,\zeta,\mathbf{Q}}P^{*\xi}P^{*\xi}B^{\xi,\xi}_{+,\zeta}-\sum_{\zeta,\mathbf{Q}}W_{Z,\mathbf{Q}}\Phi^{\text{R}}_{+,\zeta,\mathbf{Q}}P^{*\xi}Z^{\xi,\xi,\xi}_{+,\zeta}-\text{c.c.},\end{aligned} \quad (14)$$

with $\alpha/\beta=\frac{m_e/m_h}{m_e+m_h}$. The coupling elements $D_{\mathbf{Q}}$ (incoherent coupling to the exciton-exciton continuum via optical interaction), $W_{B,\mathbf{Q}}$ and $W_{Z,\mathbf{Q}}$ (incoherent coupling to the exciton-exciton continuum via Coulomb interaction) are given in Eq. (24), Eq. (25) and Eq. (26), respectively. The equations of motion of the spin-like ($\xi=\xi'$) and spin-unlike ($\xi\neq\xi'$) biexciton correlations B read:

$$\begin{aligned}\text{i}\hbar\partial_t B^{\xi,\xi'}_{\pm,\zeta} =& \left(E^{\text{XX},\xi,\xi'}_{\pm,\zeta}-\text{i}\hbar\gamma_B\right)B^{\xi,\xi'}_{\pm,\zeta}+\frac{1}{2}\left(1\pm\delta_{\xi,\xi'}\right)\sum_{\mathbf{K}}\Phi^{*\text{L}}_{\pm,\zeta,\mathbf{K}}\sum_{\mathbf{Q}}\left(S^{-1}_\pm\right)_{\mathbf{K};\mathbf{Q}}\left(\pm W_{1,\mathbf{Q},\mathbf{0}}-W_{2,\mathbf{Q},\mathbf{0}}\right)P^\xi P^{\xi'}\\ &+\frac{1}{2}\sum_{\mathbf{K}}\Phi^{*\text{L}}_{\pm,\zeta,\mathbf{K}}\sum_{\mathbf{Q}}\left(S^{-1}_\pm\right)_{\mathbf{K};\mathbf{Q}}\left(\pm W_{\text{X},1,\mathbf{Q},\mathbf{0}}-W_{\text{X},2,\mathbf{Q},\mathbf{0}}+\delta_{\xi,\xi'}\left(W_{\text{X},1,\mathbf{Q},\mathbf{0}}\mp W_{\text{X},2,\mathbf{Q},\mathbf{0}}\right)\right)P^\xi P^\xi\\ &+\frac{1}{2}\sum_{\mathbf{K}}\Phi^{*\text{L}}_{\pm,\zeta,\mathbf{K}}\sum_{\mathbf{Q}}\left(S^{-1}_\pm\right)_{\mathbf{K};\mathbf{Q}}\left(\pm W_{\text{X},1,-\mathbf{Q},\mathbf{0}}-W_{\text{X},2,-\mathbf{Q},\mathbf{0}}+\delta_{\xi,\xi'}\left(W_{\text{X},1,-\mathbf{Q},\mathbf{0}}\mp W_{\text{X},2,-\mathbf{Q},\mathbf{0}}\right)\right)P^{\xi'}P^{\xi'}.\end{aligned} \quad (15)$$

Here, γ_B is the biexcitonic dephasing, which is set as twice the excitonic nonradiative dephasing: $\gamma_B=2\gamma_{\text{nrad}}$.The equations of motion of the spin-like exciton-biexciton correlations Z read:

$$\begin{aligned}\text{i}\hbar\partial_t Z^{\xi,\xi,\xi}_{\pm,\zeta} =& \left(E^{\text{XX}}_{\pm,\zeta}-E_{1s}-\text{i}\hbar\gamma_R\right)Z^{\xi,\xi,\xi}_{\pm,\zeta}+(1\pm1)\sum_{\mathbf{K}}\Phi^{*\text{L}}_{\pm,\zeta,\mathbf{K}}\sum_{\mathbf{Q}}\left(S^{-1}_\pm\right)_{\mathbf{K};\mathbf{Q}}\left(\pm W_{1,\mathbf{Q},\mathbf{0}}-W_{2,\mathbf{Q},\mathbf{0}}\right)P^\xi N^\xi\\ &+(1\pm1)\sum_{\mathbf{K}}\Phi^{*\text{L}}_{\pm,\zeta,\mathbf{K}}\sum_{\mathbf{Q}}\left(S^{-1}_\pm\right)_{\mathbf{K};\mathbf{Q}}\left(W_{\text{X},1,\mathbf{Q},\mathbf{0}}+W_{\text{X},1,-\mathbf{Q},\mathbf{0}}-W_{\text{X},2,\mathbf{Q},\mathbf{0}}-W_{\text{X},2,-\mathbf{Q},\mathbf{0}}\right)P^\xi N^\xi,\end{aligned} \quad (16)$$

and of the spin-unlike exciton-biexciton correlations:

$$\begin{aligned}
\mathrm{i}\hbar\partial_t Z^{\xi,\xi',\xi''}_{\pm,\zeta} &= \left(E^{\mathrm{XX}}_{\pm,\zeta} - E_{1s} - \mathrm{i}\hbar\gamma_R\right) Z^{\xi,\xi',\xi''}_{\pm,\zeta} \\
&+ \frac{1}{2}\left(1-\delta_{\xi,\xi'}\delta_{\xi,\xi''}\right)\sum_{\mathbf{K}} \Phi^{*\mathrm{L}}_{\pm,\zeta,\mathbf{K}} \sum_{\mathbf{Q}} \left(S^{-1}_{\pm}\right)_{\mathbf{K};\mathbf{Q}} \left(\pm W_{1,\mathbf{Q},\mathbf{0}} - W_{2,\mathbf{Q},\mathbf{0}}\right) P^{\xi'} N^{\xi} \delta_{\xi,\xi''} \\
&+ \left(1-\delta_{\xi,\xi'}\delta_{\xi,\xi''}\right)\sum_{\mathbf{K}} \Phi^{*\mathrm{L}}_{\pm,\zeta,\mathbf{K}} \sum_{\mathbf{Q}} \left(S^{-1}_{\pm}\right)_{\mathbf{K};\mathbf{Q}} \left(\pm W_{\mathrm{X},1,\mathbf{Q},\mathbf{0}} - W_{\mathrm{X},2,\mathbf{Q},\mathbf{0}}\right) P^{\xi} N^{\xi} \delta_{\xi,\xi'} .
\end{aligned} \tag{17}$$

γ_R denotes the dephasing of the exciton-biexciton transitions, which is set as three times the excitonic nonradiative dephasing: $\gamma_R = 3\gamma_{\mathrm{nrad}}$.

We additionally include an effective phonon-assisted formation of incoherent occupations [9–11] in Eq. (14):

$$\mathrm{i}\hbar\partial_t N^{\xi}\big|_{\mathrm{phon}} = \Gamma_{\mathrm{form}} \left|P^{\xi}\right|^2 . \tag{18}$$

The effective formation rate $\Gamma_{\mathrm{form}} = 2\gamma_{\mathrm{phon}}$, where γ_{phon} is the phonon-assisted contribution to the dephasing of the excitonic transition [12], is calculated microscopically by self-consistently considering slightly detuned excitation as in Ref. [13]. For the $MoSe_2$ monolayer, we take A′, TO, LA and TA phonon modes in deformation potential approximation (monolayer $MoSe_2$) [12, 14] into account. For the GaAs MQW, we take one LO mode via Fröhlich coupling and one LA mode via deformation potential coupling [9, 15–17] into account. It turns out, that, in the $MoSe_2$ monolayer, phonon-assisted incoherent occupation formation in Eq. (18) plays a significant role and outcompetes the formation due to optical interaction in Eq. (14) even at temperatures of 5 K, while in the MWQ sample, at a temperature of 6 K, it is insignificant compared to optical formation at the applied pump powers. Since we are only interested in the dynamics within or shortly after the optical pulse, all other incoherent scattering mechanisms, e.g., phonon-assisted thermalization and intervalley scattering of excitonic occupations [9, 10, 18], Coulomb-mediated intervalley exchange [19–21] or Auger scattering [22–24], which additionally contribute to Eq. (14), are neglected.

We solve the equations of motion of the biexciton and exciton-biexciton continua in Markov approximation [25], which reduces the set of equations of motion of B and Z to the respective bound state for the numerics. Since the optical pump pulses used in this work feature a comparably long duration, we expect that non-Markovian effects play a minor role [26]. In case of the MQW sample, we assume ten systems of equations of motion, one for every optically active well, which are coupled radiatively [4], where we neglect the propagation of the pulse envelope in z-direction. Since FWHM $\cdot\, c_0 \approx 400\,\mu\mathrm{m}$ is much larger than the total width of the MQW (362 nm), propagation effects of the pulse envelope should be negligible.

Since our theory is microscopic, we only have to adjust the $1s$ excitonic energy E_{1s}, the nonradiative linewidth $\hbar\gamma_{\mathrm{nrad}}$ and the transition dipole element d^{cv} to the corresponding measured linear absorption spectrum, cf. Sec. *Linear Absorption*. All other many-body exciton-exciton matrix elements entering Eq. 6 and Eqs. 14–(17) are calculated with the excitonic wave functions $\varphi_{\mathbf{q}}$ and the two-exciton wave functions $\Phi^{\mathrm{L/R}}_{\pm,\alpha,\mathbf{Q}}$ by solving the corresponding one-exciton and two-exciton eigenvalue problem, cf. Eq. (11) and Eq. (12), respectively [27, 28], with a quantum-confined Coulomb potential $V_{\mathbf{q}}$, which takes screening due to the dielectric environment and the material itself into account, cf. Sec. *Screened Coulomb Potential*.

In Tab. SI and Tab. SII, we show the simulation parameters of the $MoSe_2$ monolayer and the GaAs MWQ sample, respectively.

Exciton-Exciton Interaction Matrix Elements

In this section, we provide the explicit expressions for the exciton-exciton matrix elements appearing in Eq. (12), Eq. (6), Eq. (14), Eq. (15), (16) and Eq. (17):

$$(S_\pm)_{\rho,\lambda,\mathbf{Q},\mu,\nu,\mathbf{K}} = \delta_{\rho,\mu}\delta_{\lambda,\nu}\delta_{\mathbf{Q},\mathbf{K}} \mp \sum_{\mathbf{q}} \varphi^*_{\rho,\mathbf{q}+\beta\mathbf{Q}}\varphi^*_{\lambda,\mathbf{q}+\mathbf{K}+\alpha\mathbf{Q}}\varphi_{\mu,\mathbf{q}+\beta\mathbf{K}}\varphi_{\nu,\mathbf{q}+\alpha\mathbf{K}+\mathbf{Q}}, \tag{19}$$

$$W_{1,\lambda,\sigma,\mathbf{Q},\nu,\eta,\mathbf{K}} = V_{\mathbf{Q}-\mathbf{K}}\sum_{\mathbf{q}}\varphi^*_{\lambda,\mathbf{q}}\left(\varphi_{\nu,\mathbf{q}+\beta(\mathbf{K}-\mathbf{Q})} - \varphi_{\nu,\mathbf{q}-\alpha(\mathbf{K}-\mathbf{Q})}\right)\sum_{\mathbf{q}'}\varphi^*_{\sigma,\mathbf{q}'}\left(\varphi_{\eta,\mathbf{q}'+\alpha(\mathbf{K}-\mathbf{Q})} - \varphi_{\eta,\mathbf{q}'-\beta(\mathbf{K}-\mathbf{Q})}\right), \tag{20}$$

$$W_{2,\lambda,\sigma,\mathbf{Q},\nu,\eta,\mathbf{K}} = \sum_{\mathbf{q},\mathbf{q}'} V_{\mathbf{q}-\mathbf{q}'+\mathbf{K}}\varphi^*_{\lambda,\mathbf{q}+\beta\mathbf{Q}}\varphi^*_{\sigma,\mathbf{q}'+\alpha\mathbf{Q}}\left(\varphi_{\nu,\mathbf{q}'-\alpha\mathbf{K}} - \varphi_{\nu,\mathbf{q}+\beta\mathbf{K}}\right)\left(\varphi_{\eta,\mathbf{q}'+\mathbf{Q}-\beta\mathbf{K}} - \varphi_{\eta,\mathbf{q}+\mathbf{Q}+\alpha\mathbf{K}}\right), \tag{21}$$

$$W^{\xi,\xi'}_{\mathrm{X},1,\mathbf{Q},\mathbf{0}} = V^{\xi,\xi'}_{\mathrm{X},\mathbf{Q}}\sum_{\mathbf{q},\mathbf{q}'}\varphi^*_{1s,\mathbf{q}+\beta\mathbf{Q}}\varphi^*_{1s,\mathbf{q}'}\varphi_{1s,\mathbf{q}+\mathbf{Q}}\varphi_{1s,\mathbf{q}'}, \tag{22}$$

$$W^{\xi,\xi'}_{\mathrm{X},2,\mathbf{Q},\mathbf{0}} = \sum_{\mathbf{q},\mathbf{q}'} V^{\xi,\xi'}_{\mathrm{X},\mathbf{q}-\mathbf{q}'+\mathbf{Q}}\varphi^*_{1s,\mathbf{q}+\beta\mathbf{Q}}\varphi^*_{1s,\mathbf{q}'-\beta\mathbf{Q}}\varphi_{1s,\mathbf{q}'-\mathbf{Q}}\varphi_{1s,\mathbf{q}}. \tag{23}$$

$$\begin{aligned} D_{\mathbf{Q}} = &\sum_{\mathbf{q}}\varphi_{\mathbf{q}+\alpha\mathbf{Q}}\varphi^*_{\mathbf{q}+\mathbf{Q}}\varphi_{\mathbf{q}+\beta\mathbf{Q}} + \sum_{\mathbf{q}}\varphi_{\mathbf{q}-\beta\mathbf{Q}}\varphi^*_{\mathbf{q}-\mathbf{Q}}\varphi_{\mathbf{q}-\alpha\mathbf{Q}} - \sum_{\mathbf{q},\mathbf{q}'}\varphi_{\mathbf{q}+\beta\mathbf{q}'}\varphi^*_{\mathbf{q}+\mathbf{Q}+\beta\mathbf{q}'}\varphi^*_{\mathbf{q}}\varphi_{\mathbf{q}+\mathbf{q}'+\beta\mathbf{Q}}\varphi_{\mathbf{q}+\alpha\mathbf{Q}+\beta\mathbf{q}'} \\ &- \sum_{\mathbf{q},\mathbf{q}'}\varphi_{\mathbf{q}+\beta\mathbf{Q}}\varphi^*_{\mathbf{q}+\mathbf{Q}+\beta\mathbf{q}'}\varphi^*_{\mathbf{q}+\mathbf{q}'-\beta\mathbf{Q}}\varphi_{\mathbf{q}+\mathbf{q}'+\beta\mathbf{Q}}\varphi_{\mathbf{q}+\alpha\mathbf{Q}}, \end{aligned} \tag{24}$$

$$\begin{aligned} W_{B,\mathbf{Q}} = &\sum_{\mathbf{K}} V_{\mathbf{K}}\sum_{\mathbf{q}}\varphi^*_{\mathbf{q}+\mathbf{Q}-\alpha\mathbf{K}}\left(\varphi^*_{\mathbf{q}-\mathbf{K}} - \varphi^*_{\mathbf{q}}\right)\varphi_{\mathbf{q}+\beta\mathbf{Q}}\varphi_{\mathbf{q}+\alpha\mathbf{Q}-\mathbf{K}}\sum_{\mathbf{q}'}\varphi_{\mathbf{q}'}\left(\varphi^*_{\mathbf{q}'-\beta\mathbf{K}} - \varphi^*_{\mathbf{q}'+\alpha\mathbf{K}}\right) \\ &+ \sum_{\mathbf{K},\mathbf{q},\mathbf{q}'} V_{\mathbf{q}-\mathbf{q}'}\varphi^*_{\mathbf{q}+\mathbf{Q}-\alpha\mathbf{K}}\left(\varphi^*_{\mathbf{q}'}\varphi^*_{\mathbf{q}-\mathbf{K}} + \varphi^*_{\mathbf{q}}\varphi^*_{\mathbf{q}'-\mathbf{K}}\right)\left(\varphi_{\mathbf{q}'-\alpha\mathbf{K}} - \varphi_{\mathbf{q}-\alpha\mathbf{K}}\right)\varphi_{\mathbf{q}+\beta\mathbf{Q}}\varphi_{\mathbf{q}-\mathbf{K}+\alpha\mathbf{Q}} \\ &- V_{\mathbf{Q}}\sum_{\mathbf{q}}\left(\varphi^*_{\mathbf{q}+\mathbf{Q}} - \varphi^*_{\mathbf{q}}\right)\varphi_{\mathbf{q}+\beta\mathbf{Q}}\sum_{\mathbf{q}'}\left(\varphi^*_{\mathbf{q}'+\beta\mathbf{Q}} - \varphi^*_{\mathbf{q}'-\alpha\mathbf{Q}}\right)\varphi_{\mathbf{q}'} \\ &- \sum_{\mathbf{q},\mathbf{q}'} V_{\mathbf{q}-\mathbf{q}'}\left(\varphi^*_{\mathbf{q}'}\varphi^*_{\mathbf{q}+\mathbf{Q}} + \varphi^*_{\mathbf{q}}\varphi^*_{\mathbf{q}'+\mathbf{Q}}\right)\left(\varphi_{\mathbf{q}'+\alpha\mathbf{Q}} - \varphi_{\mathbf{q}+\alpha\mathbf{Q}}\right)\varphi_{\mathbf{q}+\beta\mathbf{Q}} \\ &+ \sum_{\mathbf{K},\mathbf{q},\mathbf{q}'} V_{\mathbf{q}-\mathbf{q}'}\left(\varphi_{\mathbf{q}'+\beta\mathbf{K}}\varphi^*_{\mathbf{q}'+\mathbf{K}}\varphi_{\mathbf{q}'} - \varphi_{\mathbf{q}+\beta\mathbf{K}}\varphi^*_{\mathbf{q}+\mathbf{K}}\varphi_{\mathbf{q}}\right)\left(\varphi^*_{\mathbf{q}'+\mathbf{Q}+\beta\mathbf{K}} - \varphi^*_{\mathbf{q}+\mathbf{Q}+\beta\mathbf{K}}\right)\varphi^*_{\mathbf{q}+\mathbf{K}+\beta\mathbf{Q}}\varphi_{\mathbf{q}'+\alpha\mathbf{Q}} \\ &- \sum_{\mathbf{K}} V_{\mathbf{Q}+\mathbf{K}}\sum_{\mathbf{q}}\varphi_{\mathbf{q}+\beta\mathbf{K}}\varphi^*_{\mathbf{q}+\mathbf{K}}\varphi^*_{\mathbf{q}}\left(\varphi_{\mathbf{q}-\alpha\mathbf{Q}} - \varphi_{\mathbf{q}+\mathbf{K}+\beta\mathbf{Q}}\right)\sum_{\mathbf{q}'}\left(\varphi^*_{\mathbf{q}'+\mathbf{Q}+\beta\mathbf{K}} - \varphi^*_{\mathbf{q}'-\alpha\mathbf{K}}\right)\varphi^*_{\mathbf{q}'+\alpha\mathbf{Q}}, \end{aligned} \tag{25}$$

$$\begin{aligned} W_{Z,\mathbf{Q}} = &2W_{B,\mathbf{Q}} + \sum_{\mathbf{q},\mathbf{q}'} V_{\mathbf{q}-\mathbf{q}'}\left(\varphi^*_{\mathbf{q}'} - \varphi^*_{\mathbf{q}}\right)\left(\varphi^*_{\mathbf{q}+\mathbf{Q}} - \varphi^*_{\mathbf{q}'+\mathbf{Q}}\right)\varphi_{\mathbf{q}+\beta\mathbf{Q}}\varphi_{\mathbf{q}'+\alpha\mathbf{Q}} \\ &- V_{\mathbf{Q}}\sum_{\mathbf{q}}\left(\varphi^*_{\mathbf{q}+\mathbf{Q}} - \varphi^*_{\mathbf{q}}\right)\varphi_{\mathbf{q}+\beta\mathbf{Q}}\sum_{\mathbf{q}'}\left(\varphi^*_{\mathbf{q}'} - \varphi^*_{\mathbf{q}'+\mathbf{Q}}\right)\varphi_{\mathbf{q}'+\alpha\mathbf{Q}}. \end{aligned} \tag{26}$$

Optical Observable

To account for a two-pulse geometry, we apply a rotating wave approximation (RWA) and perform a spatial Fourier expansion in the exciting fields by parametrically reintroducing the center-of-mass coordinate $\mathbf{R}$ [29–31]:

$$\mathbf{E}_0(t) \to \mathbf{E}_0(t,\mathbf{R}) = \mathbf{E}_0^{10}(t)\mathrm{e}^{\mathrm{i}\mathbf{k}_1\cdot\mathbf{R}} + \mathbf{E}_0^{01}(t-\tau)\mathrm{e}^{\mathrm{i}\mathbf{k}_2\cdot\mathbf{R}}. \tag{27}$$

Here, $\mathbf{E}_0^{10}(t)$ and $\mathbf{k}_1$ as well as $\mathbf{E}_0^{01}(t-\tau)$ and $\mathbf{k}_2$ are the envelopes as well as propagation directions of the resonant parts of the incident pump and probe pulse, respectively, and τ is the delay time. The excitonic transition P, occupation

N, biexciton B and exciton-biexciton transition Z are expanded accordingly:

$$P(\mathbf{R}) = \sum_{\substack{n_1,n_2,\\ n_1+n_2=1}} \mathrm{e}^{\mathrm{i}n_1\mathbf{k}_1\cdot\mathbf{R}}\mathrm{e}^{\mathrm{i}n_2\mathbf{k}_2\cdot\mathbf{R}}P^{n_1n_2}, \tag{28}$$

$$N(\mathbf{R}) = \sum_{\substack{n_1,n_2,\\ n_1+n_2=0}} \mathrm{e}^{\mathrm{i}n_1\mathbf{k}_1\cdot\mathbf{R}}\mathrm{e}^{\mathrm{i}n_2\mathbf{k}_2\cdot\mathbf{R}}N^{n_1n_2}, \tag{29}$$

$$B(\mathbf{R}) = \sum_{\substack{n_1,n_2,\\ n_1+n_2=2}} \mathrm{e}^{\mathrm{i}n_1\mathbf{k}_1\cdot\mathbf{R}}\mathrm{e}^{\mathrm{i}n_2\mathbf{k}_2\cdot\mathbf{R}}B^{n_1n_2}, \tag{30}$$

$$Z(\mathbf{R}) = \sum_{\substack{n_1,n_2,\\ n_1+n_2=1}} \mathrm{e}^{\mathrm{i}n_1\mathbf{k}_1\cdot\mathbf{R}}\mathrm{e}^{\mathrm{i}n_2\mathbf{k}_2\cdot\mathbf{R}}Z^{n_1n_2}, \tag{31}$$

so we obtain the equations of motion for the coefficients $P^{n_1n_2}$, $N^{n_1n_2}$, $B^{n_1n_2}$ and $Z^{n_1n_2}$ by sorting after the phase factors, which reflect the optical grating created by the two incident pulses. The emerging contributions are then reduced by only considering terms up to second order in the pump field and first order in the probe field, which describes the situation of an intense pump and a weak probe pulse. The macroscopic polarization $\mathbf{P}^{\mathrm{t}}(t)$ in the direction of the probe pulse reads:

$$\mathbf{P}^{\mathrm{t}}(t) = \frac{1}{\mathcal{A}}\sum_{\mathbf{q},\xi}\varphi_{\mathbf{q}}\mathbf{d}^{vc,\xi}P^{\xi,01}, \tag{32}$$

where $P^{\xi,01}$ is the Fourier component of the excitonic transition in the direction of the probe pulse. The renormalized transmitted probe field $\mathbf{E}^{\mathrm{t}}_{\mathrm{T}}(t)$ then reads:

$$\mathbf{E}^{\mathrm{t}}_{\mathrm{T}}(t) = \mathbf{E}^{01}_{0}(t) + \frac{\mathrm{i}\omega_t}{2\epsilon_0 c_0 n_{\mathrm{ref}}}\mathbf{P}^{\mathrm{t}}(t), \tag{33}$$

and the reflected probe field $\mathbf{E}^{\mathrm{t}}_{\mathrm{R}}(t)$ reads:

$$\mathbf{E}^{\mathrm{t}}_{\mathrm{R}}(t) = \frac{\mathrm{i}\omega_t}{2\epsilon_0 c_0 n_{\mathrm{ref}}}\mathbf{P}^{\mathrm{t}}(t). \tag{34}$$

Here, ω_t is the center frequency of the probe pulse and we neglected all time derivatives on the slowly-varying parts. To relate the calculated optical fields to measured spectra, we follow Beer's law and obtain for the total probe absorption αL of a sample with thickness L [32]:

$$\alpha L = -\ln\left(\frac{I(L)}{I(0)}\right), \tag{35}$$

where $I(0) \sim |\mathbf{E}^t_0(\omega)|^2$ is the incident intensity and $I(L) \sim |\mathbf{E}^t_{\mathrm{T}}(\omega,\tau)|^2$ is the transmitted intensity.

Screened Coulomb Potential

The screened Coulomb potential $V_{\mathbf{q}}$ reads:

$$V_{\mathbf{q}} = \frac{e^2}{\mathcal{A}}\int \mathrm{d}z\,\mathrm{d}z'\;|\xi(z)|^2\,G_{\mathbf{q}}(z,z')\,|\xi(z')|^2\,, \tag{36}$$

where $\xi(z)$ is the carrier confinement wave function and $G_{\mathbf{q}}(z,z')$ is the Green's function of the Poisson equation of the corresponding sample geometry of interest. To take the influence of the dielectric environment into account, we solve the Poisson equation for a five-layer geometry, i.e., for a thin semiconductor ϵ_s with thickness d and interlayer separation h to substrate ϵ_1 and superstrate ϵ_2. Here, we explicitly take a small vacuum gap h between the thin

semiconductor and the surrounding materials into account. The corresponding Green's function reads:

$$
\begin{aligned}
G_{\mathbf{q}}(z,z') &= \frac{1}{2\epsilon_0\epsilon_s|\mathbf{q}|}\mathrm{e}^{-|\mathbf{q}||z-z'|} \\
&+ \frac{1}{2\epsilon_0\epsilon_s|\mathbf{q}|f_{\mathbf{q}}}\Big(\Big(\epsilon_{s,-}\epsilon_{2,+}\mathrm{e}^{h|\mathbf{q}|}-\epsilon_{s,+}\epsilon_{2,-}\mathrm{e}^{-h|\mathbf{q}|}\Big)\Big(\epsilon_{1,+}\epsilon_{s,-}\mathrm{e}^{h|\mathbf{q}|}-\epsilon_{1,-}\epsilon_{s,+}\mathrm{e}^{-h|\mathbf{q}|}\Big)\mathrm{e}^{-d|\mathbf{q}|}\Big(\mathrm{e}^{|\mathbf{q}|(z-z')}+\mathrm{e}^{-|\mathbf{q}|(z-z')}\Big) \\
&+ \Big(\epsilon_{s,-}\epsilon_{2,+}\mathrm{e}^{h|\mathbf{q}|}-\epsilon_{s,+}\epsilon_{2,-}\mathrm{e}^{-h|\mathbf{q}|}\Big)\Big(\epsilon_{1,+}\epsilon_{s,+}\mathrm{e}^{h|\mathbf{q}|}-\epsilon_{1,-}\epsilon_{s,-}\mathrm{e}^{-h|\mathbf{q}|}\Big)\mathrm{e}^{|\mathbf{q}|(z+z')} \\
&+ \Big(\epsilon_{s,+}\epsilon_{2,+}\mathrm{e}^{h|\mathbf{q}|}-\epsilon_{s,-}\epsilon_{2,-}\mathrm{e}^{-h|\mathbf{q}|}\Big)\Big(\epsilon_{1,+}\epsilon_{s,-}\mathrm{e}^{h|\mathbf{q}|}-\epsilon_{1,-}\epsilon_{s,+}\mathrm{e}^{-h|\mathbf{q}|}\Big)\mathrm{e}^{-|\mathbf{q}|(z+z')}\Big),
\end{aligned}
\tag{37}
$$

with

$$
\begin{aligned}
f_{\mathbf{q}} = &-\epsilon_{s,-}\epsilon_{s,-}\Big(\epsilon_{1,+}\epsilon_{2,+}\mathrm{e}^{-d|\mathbf{q}|}\mathrm{e}^{2h|\mathbf{q}|}-\epsilon_{1,-}\epsilon_{2,-}\mathrm{e}^{d|\mathbf{q}|}\mathrm{e}^{-2h|\mathbf{q}|}\Big) \\
&-2\epsilon_{s,+}\epsilon_{s,-}\left(\epsilon_1\epsilon_2-1\right)\Big(\mathrm{e}^{d|\mathbf{q}|}-\mathrm{e}^{-d|\mathbf{q}|}\Big)+\epsilon_{s,+}\epsilon_{s,+}\Big(\epsilon_{1,+}\epsilon_{2,+}\mathrm{e}^{d|\mathbf{q}|}\mathrm{e}^{2h|\mathbf{q}|}-\epsilon_{1,-}\epsilon_{2,-}\mathrm{e}^{-d|\mathbf{q}|}\mathrm{e}^{-2h|\mathbf{q}|}\Big),
\end{aligned}
\tag{38}
$$

where

$$
\epsilon_{i,\pm} = \epsilon_i \pm 1. \tag{39}
$$

For cos-confinement with $\xi(z) = \sqrt{\frac{2}{d_{\mathrm{eff}}}}\cos\Big(\frac{\pi}{d_{\mathrm{eff}}}z\Big)$, which models the charge distributions in z-direction resembling *ab-initio* results [33], the integrals read:

$$
\begin{aligned}
&\frac{4}{d_{\mathrm{eff}}^2}\int_{-\frac{d_{\mathrm{eff}}}{2}}^{\frac{d_{\mathrm{eff}}}{2}}\mathrm{d}z\,\mathrm{d}z'\,\cos^2\Big(\frac{\pi}{d_{\mathrm{eff}}}z\Big)\mathrm{e}^{-|\mathbf{q}||z-z'|}\cos^2\Big(\frac{\pi}{d_{\mathrm{eff}}}z'\Big) = \\
&\qquad\frac{1}{|\mathbf{q}|d_{\mathrm{eff}}\left(4\pi^2+|\mathbf{q}|^2d_{\mathrm{eff}}^2\right)}\left(8\pi^2+3|\mathbf{q}|^2d_{\mathrm{eff}}^2-\frac{32\pi^4\left(1-\mathrm{e}^{-|\mathbf{q}|d_{\mathrm{eff}}}\right)}{|\mathbf{q}|d_{\mathrm{eff}}\left(4\pi^2+|\mathbf{q}|^2d_{\mathrm{eff}}^2\right)}\right),
\end{aligned}
\tag{40}
$$

and

$$
\frac{4}{d_{\mathrm{eff}}^2}\int_{-\frac{d_{\mathrm{eff}}}{2}}^{\frac{d_{\mathrm{eff}}}{2}}\mathrm{d}z\,\mathrm{d}z'\,\cos^2\Big(\frac{\pi}{d_{\mathrm{eff}}}z\Big)\mathrm{e}^{\pm|\mathbf{q}|(\pm z\pm z')}\cos^2\Big(\frac{\pi}{d_{\mathrm{eff}}}z'\Big) = \left(\frac{8\pi^2\sinh\Big(\frac{|\mathbf{q}|d_{\mathrm{eff}}}{2}\Big)}{|\mathbf{q}|d_{\mathrm{eff}}\left(4\pi^2+|\mathbf{q}|^2d_{\mathrm{eff}}^2\right)}\right)^2. \tag{41}
$$

Here, d_{eff} represents the width of the carrier distribution, which in reality extends slightly beyond the actual width of the semiconductor (realistic finite deep potential well vs. ideal infinitely deep potential well).

With these ingredients, an explicit expression for the screened Coulomb potential in Eq. (36) is derived:

$$
\begin{aligned}
V_{\mathbf{q}} = &\frac{e^2}{2\mathcal{A}\epsilon_0\epsilon_s|\mathbf{q}|}\frac{1}{|\mathbf{q}|d_{\mathrm{eff}}\left(4\pi^2+|\mathbf{q}|^2d_{\mathrm{eff}}^2\right)}\left(8\pi^2+3|\mathbf{q}|^2d_{\mathrm{eff}}^2-\frac{32\pi^4\left(1-\mathrm{e}^{-|\mathbf{q}|d_{\mathrm{eff}}}\right)}{|\mathbf{q}|d_{\mathrm{eff}}\left(4\pi^2+|\mathbf{q}|^2d_{\mathrm{eff}}^2\right)}\right) \\
&+\frac{e^2}{2\mathcal{A}\epsilon_0\epsilon_s|\mathbf{q}|}\frac{1}{f_{\mathbf{q}}}\left(\frac{8\pi^2\sinh\Big(\frac{|\mathbf{q}|d_{\mathrm{eff}}}{2}\Big)}{|\mathbf{q}|d_{\mathrm{eff}}\left(4\pi^2+|\mathbf{q}|^2d_{\mathrm{eff}}^2\right)}\right)^2 \\
&\times\Big(\Big(\epsilon_{s,-}\epsilon_{2,+}\mathrm{e}^{h|\mathbf{q}|}-\epsilon_{s,+}\epsilon_{2,-}\mathrm{e}^{-h|\mathbf{q}|}\Big)\Big(\epsilon_{1,+}\epsilon_{s,-}\mathrm{e}^{h|\mathbf{q}|}-\epsilon_{1,-}\epsilon_{s,+}\mathrm{e}^{-h|\mathbf{q}|}\Big)2\mathrm{e}^{-d|\mathbf{q}|} \\
&\quad+\Big(\epsilon_{s,-}\epsilon_{2,+}\mathrm{e}^{h|\mathbf{q}|}-\epsilon_{s,+}\epsilon_{2,-}\mathrm{e}^{-h|\mathbf{q}|}\Big)\Big(\epsilon_{1,+}\epsilon_{s,+}\mathrm{e}^{h|\mathbf{q}|}-\epsilon_{1,-}\epsilon_{s,-}\mathrm{e}^{-h|\mathbf{q}|}\Big) \\
&\quad+\Big(\epsilon_{s,+}\epsilon_{2,+}\mathrm{e}^{h|\mathbf{q}|}-\epsilon_{s,-}\epsilon_{2,-}\mathrm{e}^{-h|\mathbf{q}|}\Big)\Big(\epsilon_{1,+}\epsilon_{s,-}\mathrm{e}^{h|\mathbf{q}|}-\epsilon_{1,-}\epsilon_{s,+}\mathrm{e}^{-h|\mathbf{q}|}\Big)\Big).
\end{aligned}
\tag{42}
$$

In the following, we discuss the treatment of the two different screening regimes.

Monolayer $MoSe_2$

Interlayer gap h: To not overestimate the substrate screening of a monolayer TMDC, which is crucial for obtaining realistic exciton and biexciton binding energies, we set the interlayer gap as $h = 0.3\,\mathrm{nm}$ according to Ref. [34].

Effective layer width d_{eff}*:* Small variations of the carrier distribution are negligible, i.e. we set $d_{\text{eff}} = d$.
Dielectric environment: We set $\epsilon_1 = \epsilon_2 = \epsilon_{\text{h-BN},0}$ as the static dielectric constant of bulk h-BN [33].
Material dielectric function ϵ_s*:* To model the screening at higher momenta, which is also crucial for obtaining realistic exciton and biexciton binding energies, we use the model dielectric function from Ref. [35], i.e., the dielectric constant of the $MoSe_2$ monolayer gains a momentum dependence $\epsilon_s \to \epsilon_{s,\mathbf{q}}$:

$$\epsilon_{s,\mathbf{q}} = 1 + \frac{1}{(\epsilon_s - 1)^{-1} + \alpha_{\text{TF}} \frac{|\mathbf{q}|^2}{q_{\text{TF}}^2} + \frac{\hbar^2 |\mathbf{q}|^4}{4 m_0^2 \omega_{\text{pl}}^2}}. \tag{43}$$

Here, $\epsilon_s = \epsilon_{s,\mathbf{q}=\mathbf{0}}$ is the dielectric constant of the bulk material, $q_{\text{TF}} = \sqrt{\frac{3^{\frac{1}{3}} \omega_{\text{pl}}^{\frac{2}{3}} m_0^{\frac{4}{3}}}{\hbar^2 \pi^{\frac{4}{3}} \epsilon_0^{\frac{2}{3}}}}$ is the Thomas-Fermi wave vector, ω_{pl} is the bulk plasmon frequency and m_0 is the free electron mass. α_{TF} is the fitting parameter, which is set to reproduce the effective screening $\epsilon_{\text{eff},\mathbf{q}}$, defined by:

$$\epsilon_{\text{eff},\mathbf{q}} = \frac{V_{0,\mathbf{q}}}{V_{\mathbf{q}}}, \tag{44}$$

from *ab-initio* calculations from the Computational Materials Repository (CMR) [36] best. In Eq. (44), $V_{0,\mathbf{q}}$ is the unscreened confined potential, which is obtained from $V_{\mathbf{q}}$ in Eq. (36) by setting $\epsilon_i = 1$ for all i.

GaAs QW

Interlayer gap h*:* A possible small vacuum gap between the optically active material and the surrounding layers is negligible, i.e. we set $h = 0\,\text{nm}$.
Effective layer width d_{eff}*:* We set $d_{\text{eff}} = 1.3d$ to theoretically reproduce the $1s$-$2s$ splitting measured in the linear absorption spectrum, cf. Supplementary Figure 5, which agrees well with Ref. [37]. If we set $d_{\text{eff}} = d$, we would slightly underestimate the carrier screening.
Dielectric environment: We set $\epsilon_1 = \epsilon_2 = \epsilon_{\text{GaAs},0}$ as the static dielectric constant of bulk GaAs [38].
Material dielectric function ϵ_s*:* A $\mathbf{q}$-dependent model of the material dielectric function as in the case of a $MoSe_2$ monolayer is not necessary, since the $\mathbf{q}$-dependent dielectric function of GaAs is almost constant in the momentum range relevant for exciton-exciton interaction [39].

Linear Absorption

In Supplementary Figure 4, we show the measured (grey-shaded area) and calculated (blue solid line) linear absorption of a h-BN-encapsulated $MoSe_2$ monolayer around the $1s$ excitonic energy. The measured absorption shows the absorption obtained in the co-linear excitation geometry setup. Due to strong disorder, the absorption line is

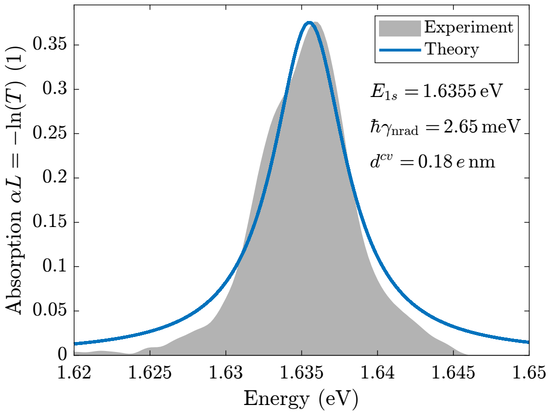

Supplementary Figure 4: Measured (grey-shaded area) and calculated (blue solid line) linear absorption of a h-BN-encapsulated monolayer $MoSe_2$ regarding the co-linear excitation geometry setup. The $1s$ excitonic energy E_{1s} and the nonradiative linewidth $\hbar\gamma_{\text{nrad}}$ are adjusted to the measured spectrum.

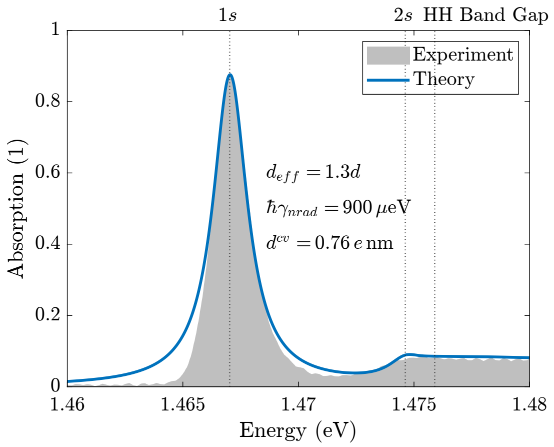

Supplementary Figure 5: Measured (grey-shaded area) and calculated (blue solid line) linear absorption of a GaAs MQW.

sensitive to the illuminated region of the sample, which differs slightly in each excitation geometry. We adjust the $1s$ excitonic energy $E_{1s} = 1.6355\,\text{eV}$, the nonradiative linewidth $\hbar\gamma_{\text{nrad}} = 2.6\,\text{meV}$ and the transition dipole moment $d^{cv} = 0.14\,e\,\text{nm}$ to the measured spectrum. The exciton-phonon scattering induced linewidth is calculated as $\hbar\gamma_{\text{phon}} = 0.22\,\text{meV}$ [12]. Therefore, the major contribution to the total nonradiative linewidth are other broadening mechanisms such as disorder [40].The radiative linewidth is calculated as $\hbar\gamma_{\text{rad}} = \frac{E_{1s}\left|\sum_{\mathbf{q}}\varphi_{\mathbf{q}}d^{cv}\right|^2}{2\hbar\epsilon_0 c_0 n_{\text{ref}}} = 0.55\,\text{meV}$.

In Supplementary Figure 5, we plot the measured (grey-shaded area) and calculated (blue solid line) linear absorption spectrum of the MQW sample with 10 radiatively coupled quantum wells [4]. The numerical values of the total nonradiative broadening $\gamma_{\text{nrad}} = 0.9\,\text{meV}$ and the electronic transition dipole moment $d^{cv} = 0.76\,e\,\text{nm}$ are adjusted to the measured spectrum. Additionally, an effective well width $d \to d_{\text{eff}} = 1.3d$ is set to obtain the correct $1s$-$2s$ energy separation. Note, that we phenomenologically set the nonradiative broadening of all higher excitonic states as twice the nonradiative broadening of the $1s$ state. We note, that the measured linear absorption displays an inhomogeneous $1s$ exciton line. Capturing this feature would involve non-Markovian disorder [41, 42], which is beyond the scope of this work.

Analytical Derivation of the Rabi Splitting

Inspired by Schäfer's textbook [5]. Excitonic equations of motion for the transition P in the coherent limit, cf. Eq. (6):

$$\mathrm{i}\hbar\partial_t P = EP - \Omega\left(1 - D\left|P\right|^2\right) + V\left|P\right|^2 P, \tag{45}$$

where E is the $1s$ excitonic energy and the excitonic Rabi energy reads:

$$\Omega = \sum_{\mathbf{q}} \varphi_{\mathbf{q}} \mathbf{d}^{cv} \cdot \mathbf{E}, \tag{46}$$

the bleaching factor reads:

$$D = 2\frac{\sum_{\mathbf{q}}\left|\varphi_{\mathbf{q}}\right|^2\varphi_{\mathbf{q}}}{\sum_{\mathbf{q}}\varphi_{\mathbf{q}}}, \tag{47}$$

and the Coulomb interaction factor reads:

$$V = 2\sum_{\mathbf{q},\mathbf{k}} V_{\mathbf{q}-\mathbf{k}}\left|\varphi_{\mathbf{q}}\right|^2\left(\varphi^*_{\mathbf{q}}\varphi_{\mathbf{k}} - \left|\varphi_{\mathbf{k}}\right|^2\right) - W, \tag{48}$$

where W is a possible red shift contribution due to biexciton continua. Fourier expanding the equations of motion in Fourier components related to an optical pump $\mathbf{E}^{1,0}$ and an optical probe $\mathbf{E}^{0,1}$ field yields the following:

$$\begin{aligned} \mathrm{i}\hbar\partial_t P^{1,0} =& EP^{1,0} - \Omega^{1,0}\left(1 - D\left|P^{1,0}\right|^2\right) \\ &+ V\left|P^{1,0}\right|^2 P^{1,0}, \end{aligned} \tag{49}$$

$$\begin{aligned} \mathrm{i}\hbar\partial_t P^{0,1} =& EP^{0,1} - \Omega^{0,1}\left(1 - D\left|P^{1,0}\right|^2\right) \\ &+ \Omega^{1,0} D P^{*1,0} P^{0,1} + \Omega^{1,0} D P^{*2,-1} P^{1,0} \\ &+ V\left(2\left|P^{1,0}\right|^2 P^{0,1} + P^{*2,-1} P^{1,0} P^{1,0}\right), \end{aligned} \tag{50}$$

$$\begin{aligned} \mathrm{i}\hbar\partial_t P^{2,-1} =& EP^{2,-1} + \Omega^{1,0} D P^{*1,0} P^{2,-1} \\ &+ \Omega^{1,0} D P^{*0,1} P^{1,0} \\ &+ V\left(2\left|P^{1,0}\right|^2 P^{2,-1} + P^{*0,1} P^{1,0} P^{1,0}\right), \end{aligned} \tag{51}$$

where we already neglected any mixed contribution in the pump equations for $P^{1,0}$ as well as any products of contributions carrying two probe excitations or more, since the probe pulse is assumed as weak. We assume plane waves and work in a rotating frame:

$$\mathbf{E}^{1,0}(t) = \mathbf{E}^{1,0}\mathrm{e}^{-\mathrm{i}\omega_0 t}, \tag{52}$$

$$\mathbf{E}^{0,1}(t) = \mathbf{E}^{0,1}\mathrm{e}^{-\mathrm{i}\omega t}, \tag{53}$$

$$P^{1,0}(t) = P^{1,0}\mathrm{e}^{-\mathrm{i}\omega_0 t}, \tag{54}$$

$$P^{0,1}(t) = P^{0,1}\mathrm{e}^{-\mathrm{i}\omega t}, \tag{55}$$

$$P^{2,-1}(t) = P^{2,-1}\mathrm{e}^{-\mathrm{i}(2\omega_0-\omega)t}. \tag{56}$$

First, we solve after the mixed transition:

$$P^{2,-1} = \frac{\Omega^{1,0} D P^{*0,1} P^{1,0} + V P^{*0,1} P^{1,0} P^{1,0}}{2\hbar\omega_0 - \hbar\omega - E - \Omega^{1,0} D P^{*1,0} - V2\left|P^{1,0}\right|^2}, \tag{57}$$

which is inserted into the equation for the probed transition $P^{0,1}$, which yields:

$$\begin{aligned} P^{0,1} =& -\Omega^{0,1}\left(1 - D\left|P^{1,0}\right|^2\right)\left(2\hbar\omega_0 - \hbar\omega - E - \Omega^{*,1,0} D P^{1,0} - V2\left|P^{1,0}\right|^2\right) \\ &\times\Bigg[\left(2\hbar\omega_0 - \hbar\omega - E - \Omega^{*,1,0} D P^{1,0} - V2\left|P^{1,0}\right|^2\right)\left(\hbar\omega - E - \Omega^{1,0} D P^{*1,0} - V2\left|P^{1,0}\right|^2\right) \\ &- \left(V P^{1,0} P^{1,0} + \Omega^{1,0} D P^{1,0}\right)\left(V P^{*1,0} P^{*1,0} + \Omega^{*,1,0} D P^{*1,0}\right)\Bigg]^{-1}, \end{aligned} \tag{58}$$

which has poles at:

$$\hbar\omega_\pm = \hbar\omega_0 \pm \sqrt{\left(\hbar\omega_0 - E\right)^2 - \left(\hbar\omega_0 - E\right)4V\left|P^{1,0}\right|^2 - 2\left(\hbar\omega_0 - E\right)\Omega^{1,0} D P^{1,0} + 3V^2\left|P^{1,0}\right|^4 + 2V\Omega^{1,0} D\left|P^{1,0}\right|^2 P^{1,0}}. \tag{59}$$

The equation for the (real) pumped transitions $P \equiv P^{1,0}$ reads:

$$VP^3 + \Omega D P^3 + \Omega D P^2 - \left(\hbar\omega_0 - E\right)P - \Omega = 0. \tag{60}$$

Case 1: $D = 0$. In the case of $D = 0$, Eq. (60) is easily solvable for resonant excitation $\hbar\omega_0 = E$:

$$P = \sqrt[3]{\frac{\Omega}{V}}, \tag{61}$$

which was also found in Ref. [43]. The Rabi splitting in Eq. (59) becomes:

$$\hbar\omega_{\pm} \equiv E_{\pm} = E \pm \sqrt{3}V^{\frac{1}{3}}\Omega^{\frac{2}{3}}, \tag{62}$$

which is sublinear in Ω, cf. Eq. (2) in the main text.

Case 2: $V = 0$. The solution of the pumped equation Eq. (60) for $V = 0$ and vanishing pump saturation ($D = 0$) reads:

$$P = -\frac{\Omega}{\hbar\omega_0 - E}, \tag{63}$$

the splitting in Eq. (59) then becomes:

$$\hbar\omega_{\pm} = \hbar\omega_0 \pm \sqrt{(\hbar\omega_0 - E)^2 + 2D\Omega^2}. \tag{64}$$

Eq. (1) in the main text is then obtained for zero detuning.

Estimating the Optical Field Strength at a Given Laser Power

The pump power in a dielectric environment with refractive index n_{ref} reads [44]:

$$P(t) = \epsilon_0 c_0 n_{\text{ref}} \int \mathrm{d}^2 r\, F(\mathbf{r}) \frac{1}{T} \int_{t-\frac{T}{2}}^{t+\frac{T}{2}} \mathrm{d}t' \, |\mathbf{E}_0(t')|^2 , \tag{65}$$

where $F(\mathbf{r})$ is the spatial intensity profile and T is the oscillation period of the center frequency. The measured time-averaged total pump power reads:

$$\bar{P} = \Gamma_{\text{rep}} \int_{-\infty}^{\infty} \mathrm{d}t\, P(t), \tag{66}$$

where Γ_{rep} is the laser repetition rate, assumed to be much smaller than the inverse temporal pulse duration σ: $\Gamma_{\text{rep}} \ll \frac{1}{\sigma}$. The intensity profile of a Gaussian beam at $z = 0$ with waist radius $w_p = \text{FWHM}_r \frac{1}{\sqrt{2\ln(2)}}$, where FWHM_r is the spatial full width at half maximum of the intensity (spot size), reads: $F_{\text{Gaussian}}(\mathbf{r}) = \mathrm{e}^{-\frac{2|\mathbf{r}|^2}{w_p^2}}$. Following Ref. [45], the pump power within the area of the probe pulse with width $w_t < w_p$ reads:

$$\bar{P}_{\text{eff}} = \frac{\int_0^{w_t} \mathrm{d}r\, \mathrm{e}^{-\frac{2r^2}{w_p^2}}}{\int_0^{\infty} \mathrm{d}r\, \mathrm{e}^{-\frac{2r^2}{w_p^2}}} \bar{P}, \tag{67}$$

which is in turn evaluated for a flat top profile, $F_{\text{FT}}(\mathbf{r}) = \Theta(w_p - |\mathbf{r}|)$, to approximate the spatial distribution of the pump pulse within the probed area:

$$\bar{P}_{\text{eff}} = \int \mathrm{d}^2 r\, F_{\text{FT}}(\mathbf{r}) \Gamma_{\text{rep}} \varepsilon_{\text{pf}}, \tag{68}$$

where we identified the pump fluence as:

$$\varepsilon_{\text{pf}} = \epsilon_0 c_0 n_{\text{ref}} \int_{-\infty}^{\infty} \mathrm{d}t\, \frac{1}{T} \int_{t-\frac{T}{2}}^{t+\frac{T}{2}} \mathrm{d}t' \, |\mathbf{E}_0(t')|^2 . \tag{69}$$

Altogether, we arrive at an equation for the pump fluence ε_{pf} at a given time-averaged total pump power $\bar{P}$:

$$\varepsilon_{\text{pf}} = \frac{1}{\Gamma_{\text{rep}} A_{\text{probe}}} \left(1 - \mathrm{e}^{-2\frac{w_t^2}{w_p^2}}\right) \bar{P}, \tag{70}$$

where the area of the probe pulse reads: $A_{\text{probe}} = \pi w_t^2$. This yields the following power-to-fluence relations for our experimental conditions:

$$\begin{aligned} 1\,\mu\text{W} &\to 1.147\,\mu\text{J}\,\text{cm}^{-2} \quad (\text{MoSe}_2\text{ monolayer}), \\ 1\,\mu\text{W} &\to 0.15\,\mu\text{J}\,\text{cm}^{-2} \quad (\text{GaAs MQW}), \end{aligned} \tag{71}$$

which is valid at the corresponding sample surface, i.e. at the vacuum/h-BN interface ($MoSe_2$) or at the vacuum/GaAs interface (GaAs MQW).

Note that, in case of the $MoSe_2$ monolayer, we defined an effective pump pulse waist radius $w_{p,\text{eff}} = \frac{\text{FWHM}_{r,\text{eff}}}{\sqrt{2\ln(2)}} > w_p$, where $\text{FWHM}_{r,\text{eff}} = \sqrt{2\text{FWHM}_r a}$ with $a = \frac{1}{2}\sqrt{\text{FWHM}_r^2 + (\text{FWHM}_r \tan(20\frac{\pi}{180}))^2}$ to account for the slightly enlarged illuminating area due to the 20° incidence angle in the experiment.

Also note, that in case of the GaAs MQW, we further have to rescale the exciting pump fluence by a factor around $\approx 1 - \frac{2}{7}$. This is necessary, since we neglect heavy-hole/light-hole coupling, which causes a redistribution of the total exciton density over heavy- and light-hole states [46].

To estimate the optical field strength, we perform the time integrals in Eq. (69) for a Gaussian-shaped optical pulse:

$$\mathbf{E}_0(t) = E_0 \frac{1}{\sqrt{2\pi}\sigma} \mathrm{e}^{-\frac{t^2}{2\sigma^2}} \frac{1}{2} \left(E_+ \mathbf{e}_+ + E_- \mathbf{e}_-\right) \mathrm{e}^{-\mathrm{i}\omega_0 t} + \text{c.c.}, \tag{72}$$

and obtain:

$$E_0 = \sqrt{\frac{4\sqrt{\pi}\sigma}{\epsilon_0 c_0 n_{\text{ref}}} \varepsilon_{\text{pf}}}. \tag{73}$$

Further, we take into account reflection losses at the vacuum/superstrate interface, which is described by the Fresnel coefficients for the amplitudes:

$$r = \frac{E_r}{E_0} = \frac{\epsilon_{\text{sup}} - \sqrt{\epsilon_{\text{sup}}}}{\epsilon_{\text{sup}} + \sqrt{\epsilon_{\text{sup}}}}, \tag{74}$$

where E_r is the reflected and E_0 is the incident amplitude, respectively. The superstrate dielectric constant in case of the h-BN-encapsulated monolayer $MoSe_2$ is $\epsilon_{\text{sup}} = \epsilon_{\text{h-BN},\parallel,\infty}$ and in case of the GaAs MQW is $\epsilon_{\text{sup}} = \epsilon_{\text{GaAs},\infty}$. Thus, the optical field strength within the corresponding semiconductor layer, which enters the excitonic Bloch equations, at a given time-averaged laser power $\bar{P}$ finally reads:

$$E_0 = (1 - r) \sqrt{\frac{4\sqrt{\pi}\sigma}{\epsilon_0 c_0 n_{\text{ref}}} \frac{1}{\Gamma_{\text{rep}} A_{\text{beam}}} \frac{\int_0^{w_t} \mathrm{d}r\, \mathrm{e}^{-\frac{2r^2}{w_p^2}}}{\int_0^{w_p} \mathrm{d}r\, \mathrm{e}^{-\frac{2r^2}{w_p^2}}} \bar{P}}. \tag{75}$$

Rescaling the Exciton-Exciton Interaction Strength in a GaAs QW

Our calculations reveal, that the action of the exciton-exciton continuum suppresses the Rabi oscillations, cf. dashed and dotted line in Supplementary Figure 6. This effect has already been observed within a different theoretical approach in Ref. [26], where Coulomb scattering have been found to efficiently suppress the Rabi oscillations of the carrier density in a strong optical field. This is a remarkable finding: The role of the exciton-exciton continuum in the excitonic-Bloch-equation approach seems to correspond already quite well to the role of Coulomb scattering in the electron-hole picture, since the total momentum-integrated excitonic density $\frac{1}{\mathcal{A}}\left(|P|^2 + \sum_{\mathbf{Q}} N_{\mathbf{Q}}\right)$ is directly comparable to the total momentum-integrated (electronic) carrier density $n = \frac{1}{\mathcal{A}}\sum_{\mathbf{k}} n_{\mathbf{k}}$. However, this is in contrast to measurements, for example those in Ref. [47] or the measurements in the present work, where multiple clear Rabi flops have been observed. Yet, when the authors in Ref. [26] added optical dressing to their theoretical description, the Rabi oscillations could be retrieved to a significant degree, resembling the dynamics of the semiconductor Bloch equations in Hartree-Fock limit without any Coulomb scattering. Therefore, to parametrically introduce the effect of optical dressing on the exciton-exciton continuum in our approach, we rescale the exciton-exciton continuum interaction strength by roughly 0.4, which mirrors the results found in Ref. [26] quite well, cf. solid line in Supplementary Figure 6.

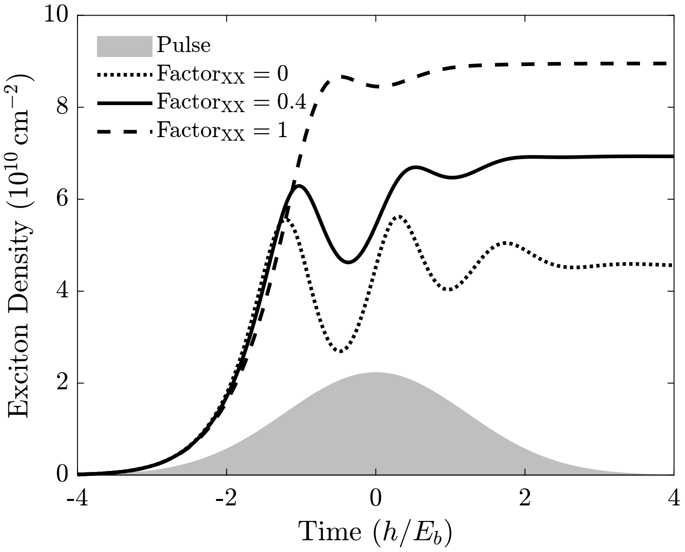

Supplementary Figure 6: Dynamics of the total $1s$ exciton density $|P|^2 + N$ for resonant excitation with 150 µW of a single GaAs quantum well for different exciton-exciton continuum scaling parameters. Here, "0" denotes no interaction with the exciton-exciton continuum, while "1" denotes full interaction strength with the (undressed) exciton-exciton continuum. The corresponding parameters are found in Tab. SII. E_b is the $1s$ exciton binding energy and h is Planck's constant.

We note, that within our excitonic approach, the explicit contributions describing optical dressing of the exciton-exciton continuum would emerge in fourth order DCT for the biexcitonic correlations B or in fifth order DCT for the exciton-biexciton correlations Z. However, expanding the excitonic equations of motion up to fifth order DCT is beyond the scope of this work.

Quantifying the Exciton-Phonon Interaction in a GaAs QW

Incoherent Exciton-Phonon Scattering: In this section, we provide microscopic calculations to quantify the impact of thermal processes in a GaAs MQW at cryogenic temperatures. The microscopic equations of motion for the exciton-phonon interactions in the excitonic picture according to Ref. [9] read:

$$\begin{aligned}\partial_t P|_{\text{X-phon}} &= -\gamma_{\text{phon}} P,\\ \partial_t N_{\mathbf{Q}}|_{\text{X-phon}} &= \Gamma^{\text{form}}_{\mathbf{Q}} |P|^2 + \sum_{\mathbf{K}} \Gamma^{\text{in}}_{\mathbf{K},\mathbf{Q}} N_{\mathbf{K}} - \sum_{\mathbf{K}} \Gamma^{\text{out}}_{\mathbf{K},\mathbf{Q}} N_{\mathbf{Q}}.\end{aligned} \tag{76}$$

The outscattering rate reads:

$$\Gamma^{\text{out}}_{\mathbf{K},\mathbf{Q}} = \frac{2\pi}{\hbar} \sum_{\pm,\alpha,q_z} \left| G^e_{\mathbf{Q}-\mathbf{K},q_z,\alpha} - G^h_{-\mathbf{Q}+\mathbf{K},q_z,\alpha} \right|^2 \left(\frac{1}{2} \pm \frac{1}{2} + n_{\pm\mathbf{Q}\mp\mathbf{K},q_z,\alpha} \right) \delta(E_{\mathbf{K}} - E_{\mathbf{Q}} \pm \hbar\omega_{\pm\mathbf{Q}\mp\mathbf{K},q_z,\alpha}), \tag{77}$$

where $\mathbf{Q}, \mathbf{K}$ are the two-dimensional excitonic center-of-mass momenta, q_z is the out-of-plane phonon momentum, α is the phonon mode, $G^{e/h}_{\mathbf{K},q_z,\alpha}$ are the exciton-phonon interaction matrix elements, $n_{\mathbf{K},q_z,\alpha}$ is the phonon occupation (Bose-Einstein distribution), $E_{\mathbf{Q}} = E + \frac{\hbar^2 \mathbf{Q}^2}{m_e + m_h}$ is the exciton dispersion and $\hbar\omega_{\mathbf{K},q_z,\alpha}$ is the phonon dispersion. The inscattering rate is obtained by:

$$\Gamma^{\text{in}}_{\mathbf{K},\mathbf{Q}} = \Gamma^{\text{out}}_{\mathbf{Q},\mathbf{K}}. \tag{78}$$

The exciton-phonon interaction matrix elements read:

$$G^e_{\mathbf{K},q_z,\alpha} = \sum_{\mathbf{q}} \varphi^*_{\mathbf{q}+\beta\mathbf{K}} \varphi_{\mathbf{q}} g^c_{\mathbf{K},q_z,\alpha} F_{q_z}, \qquad G^h_{\mathbf{K},q_z,\alpha} = \sum_{\mathbf{q}} \varphi^*_{\mathbf{q}-\alpha\mathbf{K}} \varphi_{\mathbf{q}} g^v_{\mathbf{K},q_z,\alpha} F_{q_z}, \tag{79}$$

where $\varphi_{\mathbf{q}}$ are the excitonic wave functions and F_{q_z} the confinement form factors [9]. The electron-phonon interaction potentials $g^{c/v}_{\mathbf{K},q_z,\alpha}$ are given by:

$$g^{c/v}_{\mathbf{K},q_z,\alpha} = \begin{cases} \mathrm{i}\frac{e}{\sqrt{K^2+q_z^2}}\sqrt{\frac{\hbar\omega_{\mathbf{K},q_z,\alpha}}{2\epsilon_0\mathcal{A}L}\left(\frac{1}{\epsilon_\infty}-\frac{1}{\epsilon_s}\right)}, & \alpha = \mathrm{LO}\ [17], \\ \sqrt{\frac{\hbar\sqrt{\mathbf{K}^2+q_z^2}}{2\rho_m c_{\mathrm{LA}}\mathcal{A}L}}D^{c/v}_{\mathrm{def}}, & \alpha = \mathrm{LA}\ [9], \end{cases} \tag{80}$$

i.e. we include one optical mode (LO) in Fröhlich coupling and one acoustic mode (LA) in deformation potential coupling.

Moreover, in Eq. (76), γ_{phon} is the phonon-assisted dephasing, which is obtained by self-consistently solving the following equation:

$$\gamma_{\mathrm{phon}} = \frac{1}{2}\sum_{\mathbf{K}}\Gamma^{\mathrm{out}}_{\mathbf{K},\mathbf{0}}\Big|_{E=\hbar\omega_{\mathrm{p}},\ \delta\to\mathcal{L}_{\gamma_{\mathrm{phon}}}}, \tag{81}$$

and $\Gamma^{\mathrm{form}}_{\mathbf{Q}}$ is the phonon-assisted formation rate:

$$\Gamma^{\mathrm{form}}_{\mathbf{Q}} = \Gamma^{\mathrm{in}}_{\mathbf{0},\mathbf{Q}}\big|_{E=\hbar\omega_{\mathrm{p}},\ \delta\to\mathcal{L}_{\gamma_{\mathrm{phon}}}}, \tag{82}$$

where the Dirac delta function in $\Gamma^{\mathrm{out/in}}$ is replaced by a Lorentzian, which takes into account a finite lifetime of higher correlations [48]:

$$\delta(E_{\mathbf{Q}} - \hbar\omega_p \pm \hbar\omega_{\mathbf{Q},q_z,\alpha}) \to \mathcal{L}_{\gamma_{\mathrm{phon}}}(E_{\mathbf{Q}} - \hbar\omega_p \pm \hbar\omega_{\mathbf{Q},q_z}) = \frac{1}{\pi}\frac{\hbar\gamma_{\mathrm{phon}}}{(E_{\mathbf{Q}} - \hbar\omega_{\mathrm{p}} \pm \hbar\omega_{\mathbf{Q},q_z})^2 + (\hbar\gamma_{\mathrm{phon}})^2}. \tag{83}$$

Here, ω_{p} is the center frequency of the optical field.

We note, that we treat the phonons as a bath with a fixed lattice temperature, i.e. the lattice cannot heat up due to optically excited exciton densities within our assumptions. However, we only need an order-of-magnitude estimate of the exciton-phonon scattering times, for which the bath approximation is well sufficient.

We adjust the lattice temperature of the 7.6 nm GaAs quantum well to 6 K, which reflects our measurement conditions.

Via Eq. (81), we calculate a phonon-assisted (half) linewidth of $\hbar\gamma_{\mathrm{phon}} = 8.3\,\mu\mathrm{eV}$ at a temperature of $T = 6\,\mathrm{K}$, which is roughly in line with literature values from theory and experiment [15, 49]. Here, we already observe, that exciton-phonon scattering has a negligible impact on the total measured (half) nonradiative linewidth of 0.9 meV.

In Supplementary Figure 7(left), we depict the total outscattering rates $\sum_{\mathbf{K}}\Gamma^{\mathrm{out}}_{\mathbf{K},\mathbf{Q}}$ from Eq. (77) from an initial state at $\mathbf{Q}$ integrated over all possible final states $\mathbf{K}$. In Supplementary Figure 7(right), we depict the inverse, the scattering time. We observe, that the scattering time of the interaction of an exciton with LA phonons is of the order of 10–40 ps, much longer than our experimentally observed timeframe of a few ps, which renders exciton-phonon scattering with acoustic phonons irrelevant within our measurement conditions. On the other side, the scattering time of the interaction with LO phonons is of the order of 1–2 ps. However, Coulomb-enhanced optical formation, cf. first term in Eq. (14) only induces excitonic occupations at center-of-mass momenta well below $0.5\,\mathrm{nm}^{-1}$, so that the states required for exciton-phonon scattering with optical phonons to occur are unoccupied. As a consequence, exciton-phonon scattering via optical phonons is irrelevant within our measurement conditions.

Coherent phonon oscillations: In this section, we examine a possible impact of coherent phonons on the measured oscillations in the (Ga,In)As MQW pump-probe spectra. We establish the equations of motion for the coherent phonon amplitude $D_{\mathbf{K},\alpha} = \langle b_{\mathbf{K},\alpha}\rangle + \langle b^\dagger_{-\mathbf{K},\alpha}\rangle$ [50] in the excitonic picture [51], where $b^{(\dagger)}_{\mathbf{K},\alpha}$ are the phonon annihilation (creation) operators at phonon momentum $\mathbf{K}$ and phonon mode α:

$$\partial_t^2 D_{\mathbf{K},\alpha} + \omega^2_{\mathbf{K},\alpha}D_{\mathbf{K},\alpha} = -\frac{1}{\hbar}\omega_{\mathbf{K},\alpha}\delta_{\mathbf{K},\mathbf{0}}2\mathrm{Re}\Big(g^c_{-\mathbf{K},\alpha} - g^v_{-\mathbf{K},\alpha}\Big)|P|^2. \tag{84}$$

Here, $\omega_{\mathbf{K},\alpha}$ is the frequency of the oscillating coherent phonon amplitude, $g^{c/v}_{\mathbf{K},\alpha}$ is the electron-phonon conduction/valence band interaction matrix element, and P is the coherent excitonic transition (we neglect any incoherent occupations), which acts as a source term. Since the optical field strikes the sample perpendicularly, only excitonic transitions at zero center-of-mass momentum $\mathbf{Q} = \mathbf{0}$ are induced. This translates to the fact, that coherent phonons

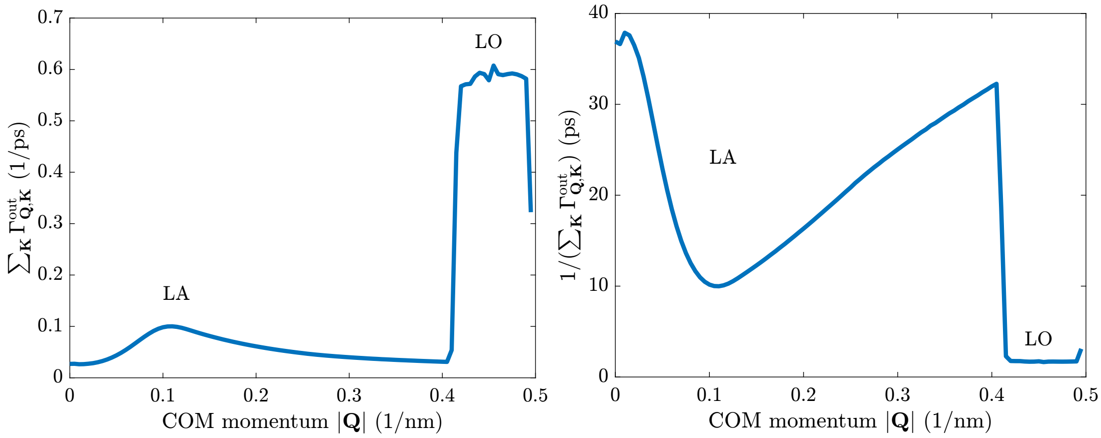

Supplementary Figure 7: Calculated outscattering rate (left) and outscattering time (right) of a 7.6 nm GaAs QW at 6 K from Eq. (77).

are also only induced at zero momentum $\mathbf{K} = \mathbf{0}$, which is encoded in the Kronecker delta on the right-hand side of Eq. (84). Since it holds: $\omega_{\mathbf{K}=\mathbf{0},\alpha} = 0$, if α = LA, and $\omega_{\mathbf{K}=\mathbf{0},\alpha} = \frac{0.0368}{\hbar}$ eV, if α = LO, only optical phonons oscillate at $\mathbf{K} = \mathbf{0}$. The corresponding period is calculated as: $T = \frac{2\pi}{\omega_{\mathbf{K}=\mathbf{0},\mathrm{LO}}} \approx 112\,\mathrm{fs}$, with optical phonon energy $\hbar\omega_{\mathbf{K}=\mathbf{0},\mathrm{LO}} = 36.8\,\mathrm{meV}$ [52]. Assuming $P(t) = \mathrm{i}\Omega$ (we work in a rotating frame with CW excitation and neglect any coherence decay, Coulomb renormalization and Pauli-blocking), where $\Omega = \frac{d^{cv}E}{\hbar}$ is the Rabi frequency with transition dipole moment d^{cv} and exciting optical field E, we can solve Eq. (84) as:

$$D_{\mathbf{K},\mathrm{LO}}(t) = -\delta_{\mathbf{K},\mathbf{0}}\frac{2}{\hbar}\mathrm{Re}\Big(g^{c}_{-\mathbf{K},\mathrm{LO}} - g^{v}_{-\mathbf{K},\mathrm{LO}}\Big)\frac{\Omega^2}{\omega_{\mathbf{K},\mathrm{LO}}}\Big(1-\cos(\omega_{\mathbf{K},\mathrm{LO}}t)\Big). \quad (85)$$

We observe, that the oscillation frequency $\omega_{\mathbf{K},\mathrm{LO}}$ of the phonon amplitude $D_{\mathbf{K},\mathrm{LO}}$ does not depend on the exciting field strength, while the oscillation frequency in the measurements, which is also reproduced by the theoretical calculations using the many-body theory in Eq. (6), Eq. (14), Eq. (15) and Eq. (16), does. Therefore, coherent phonons can be ruled out as a possible source of the measured oscillations.

Origin of the Asymmetric Rabi Splitting in a $MoSe_2$ Monolayer

First of all, the overall splitting into a repulsive and an attractive branch reflects the Stark or Rabi splitting induced by the exciting field, i.e., it reflects the light-induced dressed states. To understand the asymmetry of the splitting, we provide three calculations:

(i) In Supplementary Figure 8(left), we depict simulations, where we neglected the Coulomb renormalizations and biexcitonic effects, i.e., we take only the first line in Eq. (6) with optical blocking into account. Here, a fully symmetric Rabi splitting is obtained.

(ii) Now, if we turn on the exciton-density-induced Coulomb renormalizations, cf. the second line in Eq. (6), a significant blue shift (repulsive behavior) of the upper branch occurs, as soon as coherent $|P|^2$ or incoherent N exciton densities are present, cf. Supplementary Figure 8(middle). The spectral position of the lower branch remains relatively stable, but its strength decreases. We note, that the strong density-dependent blue shift of the upper branch is a common feature of nonlinear absorption measurements in TMDCs [53–55], while the observation of a lower branch and, hence, a splitting is more challenging due to the necessity of small linewidths.

(iii) If we finally turn on also the biexcitonic effects in the third and fourth line of Eq. (6), which result in the formation of the bound biexciton/exciton-biexciton, excitation-induced dephasing and red-shift contributions, cf. Supplementary Figure 8(right), the strong density-dependent repulsive behavior of the upper branch is slightly attenuated and accompanied by a broadening due to the biexcitonic continuum, but still exhibits a strong density-dependent overall blue shift.

Therefore, we conclude, that the upper branch is dominated by the strong density-dependent Coulomb interaction, while the lower branch is not. Hence, the latter is a more light-dominated state compared to the former. Thus, in a $MoSe_2$ monolayer, the initially symmetric light-induced dressing occurring without Coulomb interaction, is

significantly altered by the strong Coulomb interaction, which dominates over the optical interaction ($\frac{\hbar\Omega}{E_b} \sim 10^{-3}$), resulting in a strongly asymmetric Rabi splitting both in theory and experiment.

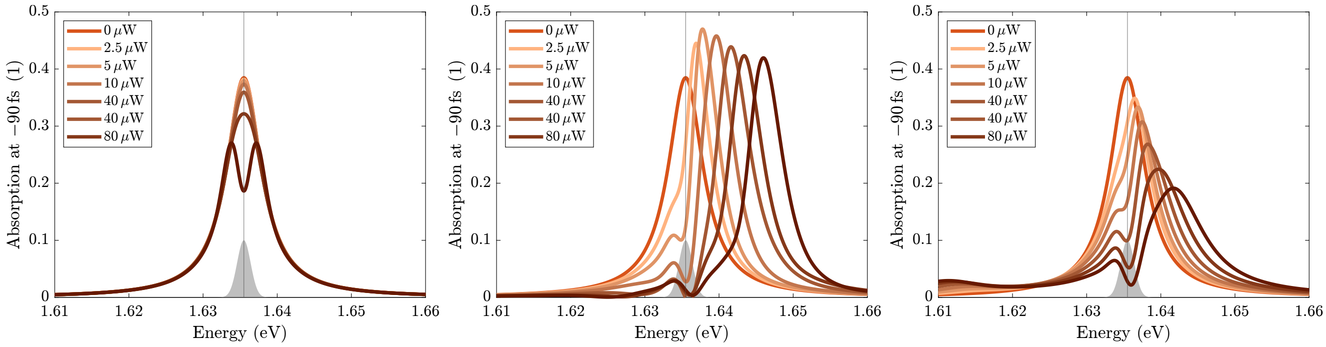

Supplementary Figure 8: Simulated pump-probe absorption of a h-BN-encapsulated $MoSe_2$ monolayer at resonant pumping. The grey-shaded area denotes the pump intensity. Left: We take only the first line in Eq. (6) into account, i.e., we neglect density-dependent Coulomb renormalization and biexcitons/exciton-biexcitons. Middle: We additionally take Coulomb renormalizations in the second line in Eq. (6) into account. Right: Full simulations, where we additionally take biexcitons and exciton-biexcitons into account as in the main manuscript, cf. lines three and four in Eq. (6).

Simulation Parameters

In Tab. SI and Tab. SII, we display the simulation parameters of the $MoSe_2$ monolayer and GaAs MQW, respectively.

TABLE SI: Parameters of the h-BN-encapsulated $MoSe_2$ monolayer.

Monolayer width d	0.6527 nm [56]
$1s$ exciton energy E_{1s}	1.6355 eV (adjusted to lin. spectrum)
$1s$ exciton binding energy	341.3 meV (calculated)
Biexciton binding energy	20.5 meV (calculated)
Electronic transition dipole moment d^{cv}	0.14 e nm (adjusted to lin. spectrum)
Lattice temperature T	5 K
Nonradiative linewidth $\hbar\gamma_{\text{nrad}}$	2.6 meV (adjusted to lin. spectrum)
Exciton-phonon scattering linewidth $\hbar\gamma_{\text{phon}}$	0.22 meV (calculated)
Radiative linewidth $\hbar\gamma_{\text{rad}}$	0.55 meV (calculated)
Phonon-assisted formation rate Γ_{form} at $\Delta = 2\,\text{meV}$	0.00124 fs^{-1} (calculated)
Static dielectric constant of bulk $MoSe_2$ $\epsilon_{s,0} = \sqrt{\epsilon_{s,\parallel,0}\epsilon_{s,\perp,0}}$	12.0474 [57]
Plasmon peak energy $\hbar\omega_{\text{pl}}$	22.0 eV [58]
Thomas-Fermi screening parameter α_{TF}	1.9 (fit to *ab-initio* results from the CMR [36])
Interlayer gap h	0.3 nm [34]
Static dielectric constant of bulk h-BN $\epsilon_{\text{h-BN},0} = \sqrt{\epsilon_{\text{h-BN},0,\parallel}\epsilon_{\text{h-BN},0,\perp}}$	4.8 [33]
Optical in-plane dielectric constant of h-BN $\epsilon_{\text{h-BN},\infty,\parallel}$	4.87 [59]
Effective electron mass m_e	$0.5m_0$ [60]
Effective hole mass m_h	$0.6m_0$ [60]
Pump pulse intensity FWHM	850 fs
Selected excitonic coefficients	
$\sum_{\mathbf{q}} \varphi_{\mathbf{q}}$	0.910
$\sum_{\mathbf{q}} \lvert\varphi_{\mathbf{q}}\rvert^2 \varphi_{\mathbf{q}}$	2.21
$\sum_{\mathbf{q},\mathbf{q}'} \varphi_{\mathbf{q}} \lvert\varphi_{\mathbf{q}'}\rvert^2 \left(\lvert\varphi_{\alpha\mathbf{q}+\beta\mathbf{q}'}\rvert^2 + \lvert\varphi_{\alpha\mathbf{q}'+\beta\mathbf{q}}\rvert^2\right)$	7.93
$\sum_{\mathbf{q},\mathbf{q}'} V_{\mathbf{q}-\mathbf{q}'} \lvert\varphi_{\mathbf{q}}\rvert^2 \left(\varphi^*_{\mathbf{q}}\varphi_{\mathbf{q}'} - \lvert\varphi_{\mathbf{q}}\rvert^2\right)$	0.396 eV
$\sum_{\mathbf{Q}} \left(-W_{1,\mathbf{Q},\mathbf{0}} - W_{2,\mathbf{Q},\mathbf{0}}\right)^* \Phi^{\text{R}}_{-,\alpha=b,\mathbf{Q}}$	0.140 eV

TABLE SII: Parameters of the GaAs MQW.

Number of optically active layers	10 (sample parameter)
Layer distance	36.2 nm (sample parameter)
Real well width d	7.6 nm (sample parameter)
Effective well width d_{eff}	$1.3d$ (adjusted to lin. spectrum)
$1s$ exciton energy E_{1s}	1.467 eV
$1s$ exciton binding energy	8.9 meV (calculated)
Electronic transition dipole moment d^{cv}	0.76 e nm (adjusted to lin. spectrum)
Lattice temperature T	6 K
Nonradiative linewidth $\hbar\gamma_{\text{nrad}}$	0.9 meV (adjusted to lin. spectrum)
Exciton-phonon scattering linewidth $\hbar\gamma_{\text{phon}}$	8.3 µeV (calculated)
Static dielectric constant of GaAs $\epsilon_{s,0}$	12.46 (5 K) [38]
Optical dielectric constant of GaAs $\epsilon_{s,\infty}$	10.58 (5 K) [38]
Effective electron mass m_e	$0.0665m_0$ [61]
Effective heavy-hole mass m_h	$0.1106m_0$ [62]
Exciton-exciton interaction scaling parameter	0.4 (fit to exp.)
Pump pulse intensity FWHM	1345 fs
Selected excitonic coefficients	
$\sum_{\mathbf{q}} \varphi_{\mathbf{q}}$	0.0585
$\sum_{\mathbf{q}} \lvert\varphi_{\mathbf{q}}\rvert^2 \varphi_{\mathbf{q}}$	32.5
$\sum_{\mathbf{q},\mathbf{q}'} \varphi_{\mathbf{q}} \lvert\varphi_{\mathbf{q}'}\rvert^2 \left(\lvert\varphi_{\alpha\mathbf{q}+\beta\mathbf{q}'}\rvert^2 + \lvert\varphi_{\alpha\mathbf{q}'+\beta\mathbf{q}}\rvert^2\right)$	117
$\sum_{\mathbf{q},\mathbf{q}'} V_{\mathbf{q}-\mathbf{q}'} \lvert\varphi_{\mathbf{q}}\rvert^2 \left(\varphi^*_{\mathbf{q}}\varphi_{\mathbf{q}'} - \lvert\varphi_{\mathbf{q}}\rvert^2\right)$	2.56 eV

[1] G. F. Mkrtchian, A. Knorr, and M. Selig, Physical Review B **100**, 125401 (2019).
[2] S. Pfalz, R. Winkler, T. Nowitzki, D. Reuter, A. Wieck, D. Hägele, and M. Oestreich, Physical Review B **71**, 165305 (2005).
[3] S. Koch, T. Meier, F. Jahnke, and P. Thomas, Applied Physics A **71**, 511 (2000).
[4] T. Stroucken, A. Knorr, P. Thomas, and S. Koch, Physical Review B **53**, 2026 (1996).
[5] W. Schäfer and M. Wegener, *Semiconductor optics and transport phenomena* (Springer Science & Business Media, 2013).
[6] R. Takayama, N. Kwong, I. Rumyantsev, M. Kuwata-Gonokami, and R. Binder, The European Physical Journal B-Condensed Matter and Complex Systems **25**, 445 (2002).
[7] A. Steinhoff, M. Florian, A. Singh, K. Tran, M. Kolarczik, S. Helmrich, A. W. Achtstein, U. Woggon, N. Owschimikow, F. Jahnke, *et al.*, Nature Physics **14**, 1199 (2018).
[8] N.-H. Kwong, J. R. Schaibley, and R. Binder, Physical Review B **104**, 245434 (2021).
[9] A. Thränhardt, S. Kuckenburg, A. Knorr, T. Meier, and S. Koch, Physical Review B **62**, 2706 (2000).
[10] M. Selig, G. Berghäuser, M. Richter, R. Bratschitsch, A. Knorr, and E. Malic, 2D Materials **5**, 035017 (2018).
[11] S. Brem, A. Ekman, D. Christiansen, F. Katsch, M. Selig, C. Robert, X. Marie, B. Urbaszek, A. Knorr, and E. Malic, Nano letters **20**, 2849 (2020).
[12] M. Selig, G. Berghäuser, A. Raja, P. Nagler, C. Schüller, T. F. Heinz, T. Korn, A. Chernikov, E. Malic, and A. Knorr, Nature communications **7**, 13279 (2016).
[13] S. Dong, S. Beaulieu, M. Selig, P. Rosenzweig, D. Christiansen, T. Pincelli, M. Dendzik, J. D. Ziegler, J. Maklar, R. P. Xian, *et al.*, Nature Communications **14**, 5057 (2023).
[14] Z. Jin, X. Li, J. T. Mullen, and K. W. Kim, Physical Review B **90**, 045422 (2014).
[15] S. Rudin and T. Reinecke, Physical Review B **65**, 121311 (2002).
[16] T.-H. Liu, J. Zhou, B. Liao, D. J. Singh, and G. Chen, Physical Review B **95**, 075206 (2017).
[17] I.-K. Oh, J. Singh, A. Thilagam, and A. Vengurlekar, Physical Review B **62**, 2045 (2000).
[18] M. Katzer, M. Selig, L. Sigl, M. Troue, J. Figueiredo, J. Kiemle, F. Sigger, U. Wurstbauer, A. W. Holleitner, and A. Knorr, Physical Review B **108**, L121102 (2023).
[19] M. Maialle, E. d. A. e Silva, and L. Sham, Physical Review B **47**, 15776 (1993).
[20] C. Lechner and U. Rössler, Physical Review B **72**, 153317 (2005).
[21] M. Selig, F. Katsch, R. Schmidt, S. Michaelis de Vasconcellos, R. Bratschitsch, E. Malic, and A. Knorr, Physical Review Research **1**, 022007 (2019).
[22] A. Steinhoff, F. Jahnke, and M. Florian, Physical Review B **104**, 155416 (2021).
[23] D. Erkensten, S. Brem, K. Wagner, R. Gillen, R. Perea-Causín, J. D. Ziegler, T. Taniguchi, K. Watanabe, J. Maultzsch, A. Chernikov, *et al.*, Physical Review B **104**, L241406 (2021).
[24] B. Han, C. Robert, E. Courtade, M. Manca, S. Shree, T. Amand, P. Renucci, T. Taniguchi, K. Watanabe, X. Marie, *et al.*, Physical Review X **8**, 031073 (2018).
[25] E. Malic and A. Knorr, *Graphene and carbon nanotubes: ultrafast optics and relaxation dynamics* (John Wiley & Sons, 2013).
[26] C. Ciuti, C. Piermarocchi, V. Savona, P. Selbmann, P. Schwendimann, and A. Quattropani, Physical Review Letters **84**, 1752 (2000).
[27] F. Katsch, M. Selig, and A. Knorr, Physical Review Letters **124**, 257402 (2020).
[28] F. Katsch, M. Selig, and A. Knorr, 2D Materials **7**, 015021 (2019).
[29] M. Lindberg, R. Binder, and S. Koch, Physical Review A **45**, 1865 (1992).
[30] R. W. Boyd and M. Sargent, JOSA B **5**, 99 (1988).
[31] G. Khitrova, P. R. Berman, and M. Sargent, JOSA B **5**, 160 (1988).
[32] P. Blood, IEEE Journal of Quantum Electronics **36**, 354 (2000).
[33] S. Latini, T. Olsen, and K. S. Thygesen, Physical Review B **92**, 245123 (2015).
[34] M. Florian, M. Hartmann, A. Steinhoff, J. Klein, A. W. Holleitner, J. J. Finley, T. O. Wehling, M. Kaniber, and C. Gies, Nano letters **18**, 2725 (2018).
[35] M. L. Trolle, T. G. Pedersen, and V. Véniard, Scientific reports **7**, 39844 (2017).
[36] K. Andersen, S. Latini, and K. S. Thygesen, Nano letters **15**, 4616 (2015).
[37] A. Liu, Physical Review B **50**, 8569 (1994).
[38] W. Moore and R. Holm, Journal of applied physics **80**, 6939 (1996).
[39] G. Cappellini, R. Del Sole, L. Reining, and F. Bechstedt, Physical Review B **47**, 9892 (1993).
[40] S. Glutsch and F. Bechstedt, Physical Review B **50**, 7733 (1994).
[41] A. Thränhardt, C. Ell, S. Mosor, G. Rupper, G. Khitrova, H. Gibbs, and S. Koch, Physical Review B **68**, 035316 (2003).
[42] R. Zimmermann, Il Nuovo Cimento D **17**, 1801 (1995).
[43] A. Knorr, T. Östreich, K. Schönhammer, R. Binder, and S. Koch, Physical Review B **49**, 14024 (1994).
[44] J.-C. Diels and W. Rudolph, *Ultrashort laser pulse phenomena* (Elsevier, 2006).
[45] D. Hayes, R. G. Hadt, J. D. Emery, A. A. Cordones, A. B. Martinson, M. L. Shelby, K. A. Fransted, P. D. Dahlberg, J. Hong, X. Zhang, *et al.*, Energy & Environmental Science **9**, 3754 (2016).
[46] R. H. Binder, M. Lindberg, A. Schuelzgen, M. Donavan, K. Wundke, H. M. Gibbs, G. Khitrova, and N. Peyghambarian, in *Physics and Simulation of Optoelectronic Devices VII*, Vol. 3625 (SPIE, 1999) pp. 80–87.

[47] A. Schülzgen, R. Binder, M. Donovan, M. Lindberg, K. Wundke, H. Gibbs, G. Khitrova, and N. Peyghambarian, Physical review letters **82**, 2346 (1999).
[48] F. Lengers, T. Kuhn, and D. Reiter, Physical Review B **101**, 155304 (2020).
[49] S. Rudin and T. Reinecke, Physical Review B **66**, 085314 (2002).
[50] A. V. Kuznetsov and C. J. Stanton, Physical review letters **73**, 3243 (1994).
[51] F. Katsch, M. Selig, A. Carmele, and A. Knorr, physica status solidi (b) **255**, 1800185 (2018).
[52] S. Rudin and T. Reinecke, Physical Review B **41**, 7713 (1990).
[53] C. Trovatello, F. Katsch, Q. Li, X. Zhu, A. Knorr, G. Cerullo, and S. Dal Conte, Nano Letters **22**, 5322 (2022).
[54] A. Rodek, T. Hahn, J. Kasprzak, T. Kazimierczuk, K. Nogajewski, K. E. Połczyńska, K. Watanabe, T. Taniguchi, T. Kuhn, P. Machnikowski, *et al.*, Nanophotonics **10**, 2717 (2021).
[55] P. D. Cunningham, A. T. Hanbicki, T. L. Reinecke, K. M. McCreary, and B. T. Jonker, Nature communications **10**, 5539 (2019).
[56] I. Kylänpää and H.-P. Komsa, Physical Review B **92**, 205418 (2015).
[57] A. Laturia, M. L. Van de Put, and W. G. Vandenberghe, npj 2D Materials and Applications **2**, 6 (2018).
[58] A. Kumar and P. Ahluwalia, Physica B: Condensed Matter **407**, 4627 (2012).
[59] Y. Cai, L. Zhang, Q. Zeng, L. Cheng, and Y. Xu, Solid state communications **141**, 262 (2007).
[60] A. Kormányos, G. Burkard, M. Gmitra, J. Fabian, V. Zólyomi, N. D. Drummond, and V. Fal'ko, 2D Materials **2**, 022001 (2015).
[61] R. Binder, I. Galbraith, and S. Koch, Physical Review B **44**, 3031 (1991).
[62] M. Bataev, M. Chukeev, M. Sharipova, P. Belov, P. Grigoryev, E. Khramtsov, I. Ignatiev, S. Eliseev, V. Lovtcius, and Y. P. Efimov, Physical Review B **106**, 085407 (2022).
